# Smart and Biomimetic 3D and 4D Printed Composite Hydrogels: Opportunities for Different Biomedical Applications

**DOI:** 10.3390/biomedicines9111537

**Published:** 2021-10-26

**Authors:** Samira Malekmohammadi, Negar Sedghi Aminabad, Amin Sabzi, Amir Zarebkohan, Mehdi Razavi, Massoud Vosough, Mahdi Bodaghi, Hajar Maleki

**Affiliations:** 1 Department of Engineering, School of Science and Technology, Nottingham Trent University, Nottingham NG11 8NS, UK; Samira_malekmohammadi@yahoo.com; 2 Department of Regenerative Medicine, Royan Institute for Stem Cell Biology and Technology, ACECR, Tehran 1665659911, Iran; masvos@royaninstitute.org; 3 Nanomedicine Research Association (NRA), Universal Scientific Education and Research Network (USERN), Tehran 1419733151, Iran; zarebkohana@tbzmed.ac.ir; 4 Department of Medical Nanotechnology, Faculty of Advanced Medical Sciences, Tabriz University of Medical Sciences, Tabriz 5166653431, Iran; neg.sedghi@gmail.com (N.S.A.); sabzia159@gmail.com (A.S.); 5 Biionix Cluster, Department of Internal Medicine, College of Medicine, University of Central Florida, Orlando, FL 32827, USA; Mehdi.Razavi@ucf.edu; 6 Department of Chemistry, Institute of Inorganic Chemistry, University of Cologne, 50939 Cologne, Germany

**Keywords:** stimuli-responsive hydrogels, 3D and 4D printing, tissue engineering, drug delivery, wound dressing, nanoelectronics

## Abstract

In recent years, smart/stimuli-responsive hydrogels have drawn tremendous attention for their varied applications, mainly in the biomedical field. These hydrogels are derived from different natural and synthetic polymers but are also composite with various organic and nano-organic fillers. The basic functions of smart hydrogels rely on their ability to change behavior; functions include mechanical, swelling, shaping, hydrophilicity, and bioactivity in response to external stimuli such as temperature, pH, magnetic field, electromagnetic radiation, and biological molecules. Depending on the final applications, smart hydrogels can be processed in different geometries and modalities to meet the complicated situations in biological media, namely, injectable hydrogels (following the sol-gel transition), colloidal nano and microgels, and three dimensional (3D) printed gel constructs. In recent decades smart hydrogels have opened a new horizon for scientists to fabricate biomimetic customized biomaterials for tissue engineering, cancer therapy, wound dressing, soft robotic actuators, and controlled release of bioactive substances/drugs. Remarkably, 4D bioprinting, a newly emerged technology/concept, aims to rationally design 3D patterned biological matrices from synthesized hydrogel-based inks with the ability to change structure under stimuli. This technology has enlarged the applicability of engineered smart hydrogels and hydrogel composites in biomedical fields. This paper aims to review stimuli-responsive hydrogels according to the kinds of external changes and t recent applications in biomedical and 4D bioprinting.

## 1. Introduction

Stereolithography, or three-dimensional (3D) printing, was first used in 1986 for printing layers or compact 3D lines or shapes by means of light [1]. This technology was developed for the rapid, versatile, and digitally-guided production of infinite amounts of objects of different scales via repetitive and well-controlled transmissions [2,3]. The most common materials used in 3D printing are ceramics [4,5], metals [6,7], polymers [8,9], and glass [10,11,12], which can all be printed from previously-developed computer-aided designs/models (CAD/CAM). From an economic point of view, 3D printing technology is highly favorable over traditional building techniques as it reduces waste in both time and material [13,14]. Importantly, the meticulous control of design parameters and synthesis procedures inhibits undesired outcomes in the late fabrication stages [15]. Due to this, 3D printing and bioprinting technologies are becoming the next global revolution, especially in the fields of pharmaceutical [16,17] and biomedical [18,19] applications. Some important applications of 3D printing include the fabrication of new protein-based biological tools (which are folded with a very high wettability similar to extracellular matrix (ECM) [20,21]), mimicking the nesting process of cancer cells [22], or tissue-engineered 3D scaffolds [23,24]. Three-dimensional (bio)printing technology enables the precise design of customized materials in a patient-specific manner, which can imitate cell communication with the ECM. The materials used are referred to as bio-inks, and choices are made based on different physicochemical properties, including biocompatibility, biodegradability, mechanical strength, gelation mechanism, electrical, thermal, optical, and response to external stimulation. Interestingly, newly introduced 4D (bio)printing technologies are built following the guidelines for 3D printing technologies. However, (bio)printing technologies are designed to fabricate functional (bio)materials responsive to internal or external stimuli, thereby changing the structure of the 3D printed biomaterials through time (the fourth dimension) [25,26,27].

Three-dimensional printing technologies are utilizing new manufacturing techniques, such as fused deposition modeling (FDM) [28], stereolithography (SLA) [29], and selective laser sintering (SLS) [30], as opposed to traditional manufacturing methods, namely CNC machining, casting, plastic injection, and so on. These 3D techniques create objects whose shape is stable and will remain stable after generation [31]. Although 4D printing technology applies the same methods as 3D printing, the printed objects are designed with advanced materials and customized designs that have the ability for structural transformation. Nevertheless, exposure to external stimuli like water, heat, current, or light is required to commence the deformation phase [32]. The types of materials utilized for the layer-upon-layer process differ between the two types of printing. In 3D printing, the final product can either be inflexible or flexible, that is, capable of retaining shape once the load is removed from it [32].

In 4D printing, the smart material changes itself under exposure to stimuli. The smart structure can be of two types—the rigid materials can be completely made from expandable materials or may be connected with expandable elements [33]. Once these expandable elements are exposed to certain stimuli, they change shape by moving or rotating, thereby transforming into a new shape. Such smart materials may be comprised of hydrogel, which can absorb a large quantity of water and expand, or polymeric materials, that can return to their original shape from a deformed state [34]. The size of the object constructed with a 3D printer depends on the size of the printer. However, in 4D printing, the size of the object can exceed the printer’s dimensional limitations. Stimuli-responsive biomaterials can be used in the concept of four-dimensional (4D) bioprinting, in which 3D printed scaffolds are designed to change over time according to one or more environment stimuli. Four-dimensional printing uses stimulus-responsive multi-media that can actively change features when subjected to suitable stimuli. From a stimulus point of view, multi-material 4D printing can be classified by their response-stimuli, such as temperature, humidity, solvents, pH, or light [35,36].

In this review, the various features and applications of 3D and 4D printing technologies used in the fabrication of multi-material objects are reviewed. This review also highlights the different advanced biomaterials available for the printing process and discusses the recent advances in 4D printing technics. The materials for 3D and 4D printing of hydrogels, including biopolymers, synthetic polymers, and nanocomposites, are principally considered. More importantly, future perspectives on 3D and 4D printing hydrogels and their importance in biomedical and bioengineering applications are discussed. Some of the common challenges and applications of 3D and 4D printed hydrogels for biomedical applications are also discussed.

## 2. Classification of Hydrogels

### 2.1. Natural Hydrogels

Based on the source of polymers, hydrogels are divided into natural (originating from plants, animals, bacteria, etc.) and synthetic [37] types.

Alginate is a linear polysaccharide harvested from brown bacteria or algae with high water solubility [38]. This biocompatible hydrogel is approved by the U. S. Food and Drug Administration (FDA) [39]. Alginate forms a hydrogel through self-assembly, using intermolecular hydrogen bonds at an acidic pH, by forming a physical hydrogel in the presence of Mg^2+^, Ca^2+^, Ba^2+^, Sr^2+^, or Al^3+^, or by utilizing sugars, including [40], mannuronic acid (M unit), guluronic acid (G unit), M-unit-rich blocks [41].

Agarose is a linear polysaccharide containing ionized sulfate groups, with physicochemical properties that completely depend on side-chain functional groups and the molecular weight of polymer chains [42,43]. Temperature highly influences the gel formation; formation must occur from 17 °C to 40 °C [44]. The gel preparation mechanism is initiated by the formation of hydrogen bonds and followed by the aggregation of double helices through the formation of bridges on discrete molecules [45]. The agarose concentration and molecular weight dictate the viscoelastic features of agarose-derived hydrogels.

Carrageenan is a red algae-derived linear and water-soluble molecule [46]. The manipulation of the position and number of sulfate groups in repeating galactose units leads to a different arrangement of polymers, and subsequently, different properties [47]. Two important types of this polymer, κ- and ι-carrageenan, can be formed by the insertion of different amounts of 3,6-anhydrogalactose in the gelation protocol [48]. The type of salts and formation time are two other vital factors, which directly affect the formation of thermo-reversible hydrogels. In contrast, a concentration of cations over a specific threshold (~0.2 M) can deteriorate the properties of the hydrogel [49]. The molecular ratio quantities of K^+^ > Ca^2+^ ≫ Na^+^ are very important in the gel formation of κ-carrageenan. This means that in the presence of high concentrations of K^+^, the rigidity and elasticity of the developed hydrogel increase, while Ca^2+^ results in stiff and brittle hydrogels [50]. In the case of ι-carrageenan, the strength of the gel increases in the order Ca^2+^ > K^+^ > Na^+^ [51].

Dextran is a highly water-soluble natural polymer with some degree of branching, which can be generated by both bacteria and chemical procedures [52,53]. The soluble form of this polymer is currently used in clinics for blood loss and hypoglycemia [46]. The special structure of this polymer (high amounts of hydroxyl group) allows them to form hydrogels using specific cross-linking molecules [54].

Cellulose has the highest abundance among other biopolymers due to its presence in numerous living organisms, from bacteria to plants [55]. The main problem regarding cellulose hydrogels is their insolubility in water and other conventional solvents [56,57]. The currently used strategy for increasing the solubility of cellulose is the etherification of its hydroxyl groups with methyl or ethyl units. There are different formulations of cellulose, including methylcellulose (MC), hydroxypropyl methylcellulose (HPMC), carboxymethylcellulose (CMC), ethylcellulose (EC), carboxyethylcellulose (CEC), and hydroxyethylcellulose (HEC), which contain physicochemical cross-linking agents in their hydrogel formulations [57]. MC is a linear and relatively flexible polysaccharide chain, with methoxy groups replaced with hydroxyl groups of different degrees [58]. This type of cellulose is adjustable within the FDA food additive criteria [59,60].

Chitosan is another polysaccharide, which is extracted by the N-deacetylation of chitin, the most abundant polymer on earth after cellulose, and structurally resembles glycosaminoglycans [61]. This polysaccharide is highly insoluble in neutral aqueous media, but after protonation of its glucosamine amine groups, it easily changes to a soluble form [62]. Chitosan is an FDA-approved polymer with high mucoadhesive features that have been explored for biomedicine [63,64,65].

Chondroitin sulfate is a pivotal element of synovial fluids and, in combination with other proteins, produces cartilage proteoglycans [66]. These hydrogels can be efficiently used for simulating cartilage interaction with other cells [67], different kinds of inflammatory cytokines like [68,69] fibroblast growth factors (FGF) [70], platelet-derived growth factors (PDGF) [71], epidermal growth factors (EGF) [70], and transforming growth factor-beta (TGF-β) [71], related to some kinds of diseases like arthritis rheumatics (AR) [72].

Hyaluronic acid can be found in almost all mammalian organs and is a non-sulfated and soluble glycosaminoglycan [72,73]. This polymer is involved in various biological activities including, proliferation, cellular migration, ECM organization, and angiogenesis and synthesis of targeted drug delivery systems [74].

Collagen is the most abundant fibrous protein [75], which is composed of five helical polypeptides enabling a great many mechanical properties [76,77]. In acidic aqueous conditions, collagen can be detected in different forms, from powder to hydrogel. In recent years, because of its ECM mimetic structure, some commercialized derivatives of these proteins were successfully designed and applied in clinical trial investigations [78,79]. One of the most famous derivatives, which has shown promising results in central nervous system (CNS) regeneration, is self-assembled peptide nanofibers (SAPNs). These nanofibers are developed from ECM proteins, including collagen, laminin, etc., which form thermosensitive hydrogels after exposure to body temperature [22].

Heparins are important polymers that can be secreted from immune cells, like mast cells, containing sulfated helical-formed glycosaminoglycan [80,81]. Due to their highly-charged negative bonds (sulfo-, hydroxyl), this polymer is the most negatively charged molecule in nature. Furthermore, like chondroitin sulfate, heparin easily interacts with different proteins and enzymes such as fibroblast growth factor (FGF), platelet factor 4, antithrombin III, fibronectin, and laminin [72,73].

Gelatin is another protein-based biological substance that is derived by heat denaturation and hydrolysis of collagen [82] and is capable of imitating biological functions [83]. The importance of this FDA-approved protein in regenerative medicine is due to the presence of arginine-glycine-aspartic acid (RGD) peptide sequences, which are vital for cell attachment to the ECM, cell proliferation, and cell differentiation [75,84,85]. However, due to its reversible thermal gelation, gelatin alone is not a good choice for regenerative medicine [86,87]. Besides human-derived protein sources, some non-human proteins are useful for application in human diseases or disorders.

Silk is a complex polymer that can be derived from different kinds of insects and worms and is used by humans as wound dressing, cloths, etc. [88,89,90]. Silk is composed of two important components, one of which is fibroin, which is a complicated mixture of hydrophobic and hydrophilic structures covered by a relatively toxic glue-like protein called sericin [91,92]. The elasto-mechanical features of silk fibroin depend on the hydrophobic/hydrophilic ratio of the proteins and their spatial orientation in different species [93,94]. In recent years, to improve the mechanical properties of silk fibroin, scientists have designed various recombinant silks [95,96,97]. Interestingly, silk proteins are successfully used for the preparation of film, foam, hydrogel, scaffold, and hybrid materials [98,99,100].

### 2.2. Polymeric Hydrogels

A polymeric gel refers to a 3D complicated network of elastic/inelastic polymer chains with or without cross-linkers that retain their solid properties and can capture a large amount of water within the network [101]. Polymeric hydrogels are composed of a moisture-retaining 3D polymeric network, which absorbs the moisture, preventing a network collapse. The internal liquid phase of such structural changes occurs between two states of matter (sol-gel transition) [102].

Based on nature, humans have used different materials and machines to construct novel hydrogels, which are better than natural primary ones. The synthetic polymeric hydrogels are categorized based on the polymer type used in their structure: (1) homopolymer-derived hydrogels, composed of only one hydrophilic polymeric monomer like PVA; (2) copolymer-derived hydrogels, prepared by crosslinking two co-monomer units (one of which should be a hydrophile); (3) multipolymer-derived hydrogels, which are prepared by crosslinking three or more co-monomer units; and (4) penetrating hydrogels, which are able to induce swelling in secondary intermeshing networks [103].

Polyacrylamide (PAAm) hydrogels are composed of acrylamide monomers (AAm) as the principal network, N, N′-methylenebisacrylamide (MBAAm) as the cross-linking agent, and ammonium persulphate (APS) as the photo/thermal initiator [104,105,106].

Poly(N-isopropylacrylamide) (PNIPAAm) hydrogels have polar side-chain peptides that are inserted into a leucine-derived polymeric chain. The characteristics of this polymer are an abrupt globular transition at 32 °C, lower critical solution temperature (LCST), hydrophilic state below the LCST, and hydrophobic state above the LCST [107]. Heating PNIPAAm to 32 °C leads to a reversible physical change, where the globule-rich polymer becomes insoluble in water. This can be reversed by changing the temperature of the environment, also called a sol-gel transition [108,109,110].

Sodium polyacrylate hydrogels are known as acrylic sodium salt polymer (ASAP), or anionic polyelectrolyte, and are negatively-charged carboxylic groups. Because of their capability to absorb water 100–1000 times more than their mass, they are also called superabsorbent polymers [111]. Used in combination with MBAAm, they become a good candidate for the formation of nanoscale-sized particles inside a hydrogel scaffold [112].

Polyethylene glycol (PEG) is a highly hydrophilic [113,114], biocompatible, highly hydrated, and FDA-approved synthetic polymer hydrogel [115,116,117]. The PEG molecules are limitedly excreted from kidneys (<30 kDa), and the only way to attain them is through the hepatobiliary system of the liver [118]. Therefore, the chain length and molecular weight of PEG dictates its application in biomedicine [119]. The melting point of PEG molecules directly depends on the molecular weight of the chain. For instance, the viscosity of low molecular weight PEG (400) is higher than heavy chains. Thus, PEG belongs to the plasticizing polymers, which, when combined or blended with other polymers, has a dramatic change in the glass transition temperature [120].

Poly(vinyl alcohol) (PVA) hydrogel is a relatively simple structure of inert polymer derived from vinyl acetate, containing hanging hydroxyl groups [121,122]. Pure PVA has been used successfully for avoiding protein adsorption and cell adhesion due to its suitable biocompatibility [123,124]. Due to crystallin formation following freezing/thawing techniques, an increase in this technique cycle leads to a reinforcing of the hydrogel’s inherent properties [125,126,127].

Unfortunately, both natural and synthetic hydrogels suffer from low mechanical strength, which is a huge drawback of hydrogel applications, especially in the biomedical field. To solve this undeniable issue, researchers have studied biologically similar structures and found that biological hydrogels are composed of different materials that increase the synergistic properties of tissues or hydrogels [127,128]. These kinds of materials are called composites, and one great category is nanocomposites. The physical basis of their strengthening behavior arises from the mechanic quantum where the different features of nanoparticles are dispersed in a matrix that reinforces the mechanical properties of the composite, including grain boundaries, tilt, interfacial adsorptive and resorptive energy, and dispersed particles. Therefore, modulating the ratio of different materials results in different properties, effectively used for tailoring the properties of hydrogels.

### 2.3. Interpenetrating Polymer Networks (IPN)

Interpenetrating polymer networks (IPN) are a combined mixture of cross-linked polymers [128], which are located in the existence of other crosslinked networks. The only way to separate such designed networks is by breaking the crosslinked locking system [129,130]. IPN hydrogels are characterized by the presence of a single network in the second swollen hydrogel or the synthesis of two different polymers in the mutual media at the same time [131,132]. One of the most interesting types of IPNs is the double-network (DN) hydrogels, in which Gong et al. [133] showed nonlinear enhancement of the mechanical properties [134,135,136]. DN hydrogels contain strong ionic crosslinked hydrogel interpenetrated by a soft and ductile polymer, which results in high water content and excellent fracture strength [137,138,139]. It is noteworthy to mention that using the nanoparticle-containing hydrogels has shown profoundly unique properties in the hydrogel structure. As mentioned above, the bottleneck of hydrogels is their low mechanical strength and Young and stress modules, but the insertion of organic or inorganic particles, especially nanoparticles due to their unusual features, can resolve these issues. Unusual properties, which can only be found in the nanoscale, including the lattice parameter, curvature, thermodynamic features, high surface to volume ratio, high entropy, self-healing features, and quantum behavior by themselves and in composite with other materials, can change the hydrogel properties, entirely using physical or chemical adding routes [140,141,142]. Given the aforementioned features, the type of nanoparticles like metallic, ceramic, silica, magnetic, and carbon-based and layered NPs all show different quantum behaviors [143]. In addition, one of the most important features of nanoparticles for reinforcing hydrogels is their biocompatibility, which must be considered in the design and synthesis of nanocomposites.

Furthermore, the type of interactions between the supramolecular structures is another crucial parameter, which strongly affects the hydrogel network’s mechanical strength, including hydrogen bonding, ionic interactions, metal coordination, and π–π stacking. Using these kinds of interactions in the nature of hydrogels increases the outstanding properties, in addition to the above-mentioned features such as bio-adhesion [144,145], stimuli-responsiveness [146,147,148], self-healing [149,150,151], adaptability [152], and molecular recognition [153,154,155].

## 3. Classification of Smart Hydrogels

As described previously, various criteria have been considered for developing suitable hydrogel-based inks for printing, including the type of polymerization processes used for the creation of homo- and co-polymers, crystallinity of monomers, the charged nature of polymers, source of polymers, size of particles, and crosslinking type [156,157,158].

### 3.1. Stimuli-Responsive Hydrogels

Stimuli-responsive hydrogels are characterized by changing some properties or functions like hydrophobicity, hydrophilicity, shape, color, and sol/gel transition speed of the hydrogel matrix [159]. In fact, smart materials are a novel class of cleverly engineered substances, which are able to change their structures in response to various types of stimuli, like light [160,161], mechanical stimulus [162,163], temperature [164], biological molecules such as glucose [165], pH [166,167], and enzymes [168,169].

### 3.2. Thermo-Responsive Hydrogels

The rapid or slow change of the matrix volume in thermo-responsive hydrogels, in response to heating, can lead to swelling, shrinking, changes in solubility, conformation alteration, and phase transition [170]. According to the behavior of polymers, there are two main types of thermo-responsive polymers, including lower critical solution temperature (LCST) or upper critical solution temperature (UCST). In the former case, the dependency of the polymer to heat changes is nonlinear, and at the LCST point, the solubility rapidly changes from a gel to a solution or vice versa. The main mechanism of this transition is the interconnections that happen between polymer chains and neighboring molecules. However, dropping the temperature of the LCST results in hydrogen bonding between the polymer chains with hydrophilic particles and leads to increased solubility of the hydrogel. With an increase in the temperature, hydrogen bonds become weaker, while the abovementioned interactions by hydrophobic entities become stronger. Consequently, shrinkage and phase transitions occur when the solubility of polymer decreases [170]. From a thermodynamic point of view, entropy is dominantly a phenomenon at the LCST. In fact, the transition of sol-gel processes increase with enthalpy (ΔH > 0) and are demonstrated as the cleavage of hydrogen bonding between polymer and water molecules, concurrently with an increase in the entropy of the system (ΔS > 0). Solvation of the polymer leads to inhibition of water molecule mobility, and afterward, the entropy of the system is reduced [171,172]. A less likely process, in UCST systems, occurs when an increase in temperature elevates the solubility of the matrix leading to the gel–sol transition. Due to the lower phase transition (<25 °C), these kinds of materials are not suitable for biomedical applications [173]. Of course, another pivotal factor, which dictates the applicability of LCST or UCST is the molecular weight (MW) of such materials, which directly affects their hydrogen bond formation capability [174]. Generally, LCST 30–37 °C is suitable for sol-gel transitions in physiological applications. Spontaneous micelle formation capability is a beneficial characteristic in the biological environment, so temperatures under the LCST result in the formation of smaller-sized, organized micelles in the body. These micelle cores, in higher temperatures, cause the formation of inter-hydrophobic bridges. These interactions are the main forces in gelation processes, which are very important for various biochemical phenomena [159,175].

### 3.3. pH-Sensitive Hydrogels

pH-responsive hydrogels, indeed, belong to thermosensitive hydrogels, and their structure is altered when pH is changed. Due to the protonation and deprotonation nature of amines in the structure of most macromolecules, we can imagine that pH-responsive polymers are polyacids (polyanions) or polybasics (polycations). Obviously, above the pKa of polyacids, polyanions are deprotonated. The conformation of polymers is changed due to a change in their ionization, with a subsequent increase in solubility. Interestingly, ionization improves the swelling behavior of the hydrogel. This means that basic polymers, at an alkaline pH, are slightly hydrated [176,177]. In contrast, acidic entities in the same media are negatively charged and have an increased dissociation rate. Clearly, dissociation results in the formation of more free ions, and subsequently, osmotic pressure increases in the hydrogel. Obviously, this osmotic pressure absorbs more water into the hydrogel structure, and this swelling causes the opening of the hydrogel pores allowing the drug molecules to release freely outside the hydrogel. Therefore, using this mechanism for specific drug release in the stomach and intestine is accessible [176]. Similar to LCST in thermosensitive polymers, changing the pH of polyelectrolyte from the critical or transition pH* zone will significantly change the hydrogel properties [178]. There are two main routes for the regulation of the pH* value in pH-sensitive smart hydrogels, including the reaction of pH-sensitive polymers with other polyacids and/or adding acidic or basic entities to the polymer backbone. Using the hydrophobic/hydrophilic structure to shift the pH* is another alternative approach [178]. In the case of cancer, there is a critical fact that efficiently influences the behavior of pH-sensitive hydrogels, which is unfortunately ignored or misconstrued in studies. One must keep in mind that the tumor microenvironment (TME) is not highly acidic, and in most cases, its pH remains the same as normal tissues [179,180]. Generally, in the worst-case scenario, the pH of the TME is changed by only one unit (pH value is 6.5–7.4). Therefore, the pH-sensitive hydrogel, in these cases, a tiny change in the pH changes the structure of the hydrogel to release the therapeutic or bio-imaging agents from the hydrogel. Unfortunately, most pH-sensitive polymers currently used are not sensitive to this amount of alteration in pH but are suitable for releasing the therapeutics intracellularly in endosomes (5.5–6) or lysosomes (4.5–5.5) as a drug carrier [181,182]. For instance, a well-established pH-sensitive polymer studied widely is PAAc, poly (sulfonic acid), and sulfonamide-derived vinyl monomers. The greatest issue in the first two groups of polymers is their wide-ranging pH sensitivity [177]. Therefore, adding sulfonamides to their matrices help them to sharpen their sensitivity for biomedical applications [183]. Other polymers with protonation ability and reaction with protons are poly (2-aminoethyl methacrylate) (PMAA), poly (N, N’-dimethyl aminoethylmethacrylate) (PDMAEMA), or poly (ethylene imine) (PEI), which contain alkaline moieties and can act as proton sponges (scavenging the H^+^ like a sponge, which absorbs the water) in acidic environments. Furthermore, pyridine-based polymers, like imidazole-based moieties, are another type of pH-sensitive material used in biomedical applications [183].

Moreover, polyanionic smart hydrogels, due to their collapsed form, in the very acidic environment (like the stomach), are currently used for protecting pH-sensitive therapeutic agents (destructed by low pH). After reaching the intestine, these hydrogels start to swell and release their cargo [184,185,186]. Poly(methacrylic acid) (PMAAc) monomers, in combination with n-butyl methacrylate (nBMA) and tert-butyl methacrylate, are one of the most famous pH protective agents, which are used for oral drug delivery of different toxic chemotherapeutic drugs [187].

### 3.4. Photo-Sensitive Hydrogels

Photosensitive hydrogels are another intelligent development in the material field, which can be used in vitro and in vivo. Generally, photosensitive hydrogels, which are developed for biomedical applications, are sensitive to visible light, near-infrared radiation (NIR), and UV stimuli [188]. The rational reason for using only these three wavelengths among the infinite electromagnetic spectrum is their safety, such as very low penetration power into the body. Given the critical issues, they can absorb and emit different spectra, respond to increasing temperatures, and interact with different kinds of hydrophobic and hydrophilic polymers, analogously with other stimulus-sensitive modules. One of the most exciting usages of NIR, as a tiny portion of electromagnetic spectra, is producing heat and ROS after interaction with specific materials, named photothermal therapy (PTT) and photodynamic therapy (PDT), which are the future candidate for chemotherapy. Noteworthy, PTT is used with some nanoparticles, which are capable of converting near-infra-red (NIR) electromagnetic spectra to heat, while PDT is a technique used with some molecules named photosensitizers (PS) for creating reactive oxygen species (ROS) [189,190,191]. Using ionizable agents under the influence of irradiation, by increasing the osmotic pressure of the intra-matrix hydrogel in combination with other materials, can be used for specific drug delivery systems [192,193,194]. Incorporation of chromophores into the matrix of hydrogels, changing a specific feature of the hydrogel, can be used for the early detection of cancer. Furthermore, using a photosensitive crosslinker in the hydrogel can detect problematic agricultural products in an early stage to reduce water and energy waste on diseased plants. Moreover, we can design and develop in situ crosslinking gel formation for biomedical printing technology [195]. Recently developed photosensitive hydrogels by Pinto et al. can arrest cancer cell proliferation by releasing CO as a therapeutic agent instead of toxic chemotherapeutic agents [196,197].

### 3.5. Hydrogels Sensitive to a Magnetic Field

In continuation of introducing the peculiar properties of nanomaterials, compared to their bulk counterparts, developing superparamagnetic nanomaterials from non-magnetic bulk materials is another outcome of nanoparticulation of materials. In the case of nanoparticles, by decreasing the scale of nanoparticles, the magnetic fields (magnetic momentum) in the crystals decrease to a minimum, which for every element has a critical diameter. Therefore, using these materials, hydrogels can be applicable for mimicking biomedical phenomena [198]. Interestingly, superparamagnetic iron oxide nanoparticles, SPIONs, one of the well-studied nanoparticles under high-frequency magnetic fields (HFMF), lead to increasing the temperature of solution or hydrogel, which is used to disperse it. This strategy is a multifunctional approach for the controlled release of drugs using alternating magnetic fields (AMF), magnetothermal eradication or apoptosis induction in cancer cells as a therapeutic route [199,200], and producing combinatorial systems, which benefit from magnetic-sensitive materials in the thermosensitive hydrogels for biomedical functions [201]. However, the thermal approach for killing cancer cells, due to the various cancer cell-resistant therapies, different signaling pathways, types of cancers, and different mechanisms of cell death, should be carefully chosen for avoiding unwanted side effects [202,203]. Generally, inducing cell death (apoptotic) following suitable parameters of magnetic hyperthermia is the best option. As mentioned earlier, combining chemotherapy drugs into magnetic-sensitive hydrogels is another cleverly designed approach. An example for using these strategies is a SPION-loaded thermosensitive polymeric hydrogel with PEGMMA backbone of different molecular weights crosslinked by various ratios of tetra (ethylene glycol) dimethacrylate (TEGDMA) or poly (ethylene glycol) dimethacrylate (PEGDMA), and finding a suitable swelling capacity over the LCST temperature. Exposure of these hydrogel systems to a controllable AMF causes an elevated temperature inside the hydrogel in response to the magnetic field and, therefore, leads to a change in the hydrogel matrices. This smart design simultaneously uses the thermal ablation of cancer cells in a medium thermal stimulus (42-45 °C) with proper chemotherapeutic drug release [204].

### 3.6. Biological Factors

Biological factors can also be considered as a stimulus for changing the hydrogel behaviors, namely to change shrinking, swelling, and decomposition [173]. There are three main stimulus-activating parameters, including the presence of enzymes, antibodies, and some biochemical agents like glucose. One of the most famous examples is insulin-releasing hydrogels in the presence of high amounts of glucose, which are characterized by glucose oxidase (GOX) and followed by pH-sensitive polymers. The principal mechanism of action in this device is the production of acid and H_2_O_2_ by polymer-attached GX enzymes, after which cationic groups are ionized by a reduced pH resulting in hydrogel swelling, and finally, insulin release out of the hydrogel [205,206]. Importantly, enzymes are the vital parts of all cells in normal and pathological situations. But the increase in function or amounts of essential enzymes in diseased sites can be a flag for identifying and treating diseases [207]. One of the fascinating findings of cancer biology in the last decades is the identification of increased levels of ECM-remodeling enzymes, which is called metalloproteinase (MMP), in cancer and other diseases [208]. Important criteria for using these enzyme-dependent strategies are the presence of enzyme substrates (unique amino acid sequence) in the hydrogel, accessibility of the substrate to the enzyme, and the cleavage reaction causing a change in the hydrogel polymeric network decomposition for drug release. Overall, the MMPs used in the matrix of hydrogels are categorized into collagenases, gelatinases, stromelysins, and cell membrane-associated MMPs [209,210].

These enzymes belong to morphogenesis in the embryogenesis step of life and are involved in differentiation, proliferation, survival, etc., which in some disorders (like cancer) remodels the ECM, increases their own size, and makes them highly activated [211,212]. Therefore, using MMP-sensitive biological substrates (GGGPQG↓IWGQGK) in combination with other smart hydrogels and bio-inks can be utilized for the synthesis of complicated hydrogels, which release their cargo in specific conditions and lead to significant inhibition of off-target phenomena [213].

β-mannanase, a small intestine-specific enzyme, is another example with a biological stimulus, the backbone of synthesized hydrogels using natural glucomannan, and decomposition that occurs by degradation of glycosidic bonds. These smart hydrogels are based on interactions between antigen-antibody; they non-covalently attach antigen and antibody and covalently interact to polymeric systems at the same time. When the concentration of free antigens increases, the stable network of antigen-polymer and the polymer-polymer structure of hydrogels are weakened by the free antigen effect on the antibodies grafted to the polymer. This reciprocal behavior loses the polymer-polymer interaction and leads to improved permeation and swelling of the hydrogel [214].

### 3.7. Moisture-Responsive Hydrogels

Because of the hydrophilic nature of hydrogels, 4D-configurable structures can use their own swelling and deswelling properties in response to environmental humidity. The crystalline copolymer poly(ethylene glycol) (PEG) and poly(tetramethylene glycol) (PTMG) have been developed as a 4D-configurable hygroscopic robot [215]. The active or moisture-sensitive layer of this robot was made from the PEG–PTMG copolymer, while its inactive layer was fabricated from beeswax. Cleverly, the hydrogen bonds of copolymers rapidly cause hygroscopic expansion of the copolymer, resulting during the humidification and dehumidification processes. Formation and dissociation of the hydrogen bonds in the copolymer lead to a fast-hygroscopic expansion of the copolymer towards the inactive layer side.

Furthermore, designing an anisotropic composite hydrogel soft N, N-dimethylacrylamide-based matrix, and stiff nano-fibrillated cellulose (NFC) is another smart hydrogel used by changing the moisture of the environment [215].

## 4. 3D Printing of Hydrogels

**Figure 1:**
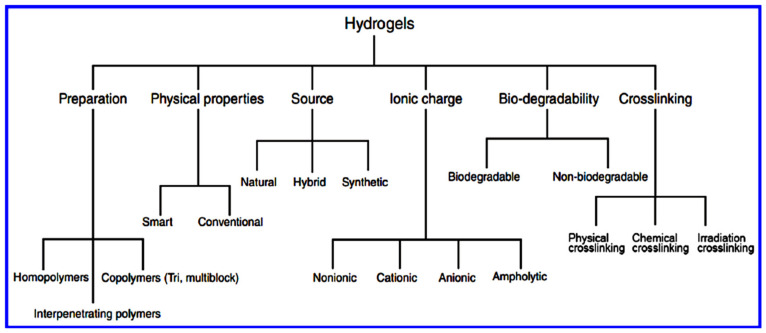
Classification of polymer hydrogels [220]. The important parameters, which should be considered based on the problem-solving plan. Copyright 2016 John Wiley@Sons. Reprinted with permission from ref. [220].

Almost all mammalian cells lie in a complicated stroma, called the extracellular matrix (ECM), which contains different kinds of proteins, glycosaminoglycans, and other soluble molecules. Oxygen and nutrients come from the arterial side of capillaries into this medium, and CO_2_ and wastes are collected from the venous end of capillaries. Furthermore, the ECM plays a pivotal role in cell migration, proliferation, differentiation, and important signaling cues to the cell [216,217]. Scientist efforts over the last century indicate that 2D media is not suitable for the assessment of cells in real-life aspects. The main logical reason behind this statement arises from the highly complicated effects of cell architects (e.g., orientation, cell-cell interaction, cell-extracellular matrix (ECM), etc.) on their behavior in response to internal/external stimuli. Indeed, one of the most important causes of failure of bench-to-bedside translation is differences in cell architects and their crosstalk in 2D compared to 3D models. For example, in the case of cancer drug delivery studies, preliminary results suggest studies be carried out on in vitro 3D tumor models due to complicated interactions between cells in 3D compared to 2D cell cultures [218]. Thus, in the last decades, the fabrication of 3D media can recapitulate the in vivo physico/biochemical and dynamic structures using new technologies [219]. However, one should keep in mind the important parameters involved in hydrogel synthesis (Figure 1). Obviously, parameter choice depends on the application of the hydrogel. For example, in the case of biomedical applications, the most important parameters are biocompatibility, biodegradability, strength, preparation method, and crosslinker; these and other parameters should be adjusted to the given hydrogel.

It seems that the ideal scaffold should meet several important requirements, such as highly porous 3D architecture with proper pore size, pore interconnectivity (which is crucial for cell growth), migration, transport of nutrients, wastes, and signaling molecules [221,222]. The scaffold’s biocompatibility, biodegradability, and physical properties of scaffold materials such as stiffness, pattern, flexibility, tailor ability, etc., are important. Moreover, another important factor in the design and development of novel scaffolds is the incorporation of physico/chemical signals necessary for the attachment of cells or induction of specific functions [222]. Finally, the supply and transition capability of specific mechanical properties such as strength, toughness, elastic stiffness, etc., for specific cell types that play the role of physical support for surrounding tissues [222,223]. In fact, in designing and developing 3D printing ink, indisputable physicochemical properties of used materials are rheological features for printability (yield stress, viscosity, shear-thinning) and cross-linking formation mechanisms (physical or chemical). It was shown that in physical hydrogels, using salt solution (CaCl_2_ for alginate), the structural integrity of the 3D scaffold is maintained [224,225]. Furthermore, changing the scaffold synthesis parameters like nozzle type, viscosity of solution by alteration of molecular weight, concentration of the polymer, extent of crosslinking, and ink composition, used for changing the mechanical properties of the scaffold [226,227]. The main drawbacks of the physical cross-linking route are that they usually result in mechanically unstable constructs. Instead, when the polymer precursors of hydrogels are functionalized by some photopolymerizable functional groups, such as acrylate, diacrylate, methacrylate, and methacrylamide, they can be used for chemical cross-linking after the 3D printing of hydrogel ink to make it mechanically more stable. In this regard, some single and dual component ink materials like poly(ethylene glycol) dimethacrylate (PEGMA) [228], gelatin-methacrylamide (GelMA) [229], hyaluronic acid/dextran methacrylate [230], and poly(ethylene glycol) diacrylate (PEGDA)/alginate [231], have been used for this purpose.

Besides biocompatibility, another important feature of bio-inks in 3D printing is its printability, which is relevant to its rheological properties and cross-linking mechanisms. Obviously, the properties of hydrogel precursors (polymers), including structure, composition, and processability, strongly depend on the concentration of polymers and molecular weight of the bio-ink. In addition, possible added components or fillers, like nanoparticles, are very important factors as well. Generally, rheology is referred to as the permanent or temporary change in the structure of materials in response to specific external stress. Shear stress, yield stress, and viscosity are the main parameters of printability in the 3D printing process. Resistance of a fluid to flow is defined as viscosity, which depends on the molecular weights and concentration of the precursor, while shear stress, which acts vice versa of viscosity, is crucial for injectability, extrudability, and printability of bio-inks. Other important factors for printability are the absence or presence of crosslinking, 3D bioprinting parameters such as nozzle diameter, dispensing speed, bioprinting speed, and extrusion pressure. Moreover, cell viability and biocompatibility are important cellular behaviors like adhesion, proliferation, signaling, and migration, which are necessary for the viability of cells, are other essential characteristics. The mechanical integrity of printed constructs and biodegradability of bio-inks and printed constructs are principal for the promotion of ECM production, bio/physicochemical cues like proteins, metabolites, drugs, and cytokines, which have a direct influence on cell fate [232,233].

### 4.1. Biomaterial Ink Selection

So far, an extensive range of materials has been developed as inks for the 3D printing of hydrogel, including natural polymers (alginate, gelatin, silk, etc.), synthetic polymers (PAA, PEG, polytetrafluoroethylene (PTFE)), biodegradable thermoplastic polymers (polycaprolactone (PCL) [234], non-degradable polymers (polyetheretherketone (PEEK)) [231], polylactide-co-glycoside (PLGA) [235], are resins (used in lithography) [236]. The abovementioned polymers are also composite with other materials like glasses, ceramics, and metals and have been introduced as novel inks due to their enforced desirable features. Overall, five superfamilies of bio-inks will be briefly discussed in the next section.

### 4.2. Single Component Hydrogel Inks

These materials are composed of only one natural or synthetic element or hydrogel precursor [237]. These kinds of gels are composed of one type of material but with different origins. The most dominant example of this category is self-assembled peptide nanofibers (SAPNS) [238,239]. The prototype of the mentioned hydrogels is RADA-16, composed of complementary peptide sequences derived from the extracellular matrix (ECM), such as collagen, laminin, fibronectin, etc. [240]. The aforementioned complementary peptides, after exposure to the human biological environment, turn into a peptide nanofiber state in a self-assemble manner, immediately based on a change in temperature and ionic composition [241]. Noteworthily, these single-component hydrogels have successfully been used, especially for curing physically damaged central nervous system (CNS) lesions like spinal cord and brain injuries [242,243].

### 4.3. IPN Hydrogel Inks

IPN hydrogels are complex structures in which the elements of the hydrogel are physically interlaced polymeric networks [244]. These inks show higher durability and strength compared to single-component hydrogel inks [245]. For instance, Laura Pescosolido et al. developed a novel semi-interpenetrating network (semi-IPN) using hyaluronic acid and hydroxyethyl-methacrylate-derivatized dextran (dex-HEMA). The suitable rheological properties, proper mechanical features, and excellent bio-activity are the main advantages of these synthesized constructs [234]. In another work, Park and coworkers, using a mix of silk and gelatin, created a novel non-toxic, biocompatible, and improved mechanical hydrogel [246].

### 4.4. Super-Molecular Hydrogel Inks

These bio-inks are used where the frequent deformation and loading of the hydrogel is needed for self-heal, self-repair, and survival of mechanical integrity [247]. An innovative host-guest supramolecule (HGSM) technology has been developed according to the inclusion reaction between cyclodextrin (CD) and adamantane (Ad). These matrices are covalently merged into a cross-linked network composed of physical noncovalent bonds [248]. The dominant features of these bio-inks are enhanced mechanical strength, self-healing capability, excellent biocompatibility, and 3D printability as ink materials [246].

## 5. 4D Printing of Hydrogels

In 2013, Tibbits and colleagues added time to 3D printing technology as the 4^th^ dimension [249,250]. Since then, 4D printing has turned into an exciting novel field for different applications. The main concept in this technology is the changing of the shape of bio-ink, over time, after exposure to an external stimulus without any permanent changes in the printed object. It should be kept in mind that there is no universal definition for 4D printing; instead, researchers usually use the Miao definition mentioned earlier. The 4D materials can also be called shape-memory materials, dielectric elastomers, liquid crystal elastomers, photosensitive polymers, and other types of stimuli-responsive hydrogels [251]. Generally, in 4D hydrogel printing technology, smart materials are incorporated along with non-smart or non-responsive materials [25]. This means that, following impregnation of the printed constructed object in a specific solvent, the hydrogel segments swell and form an asymmetric shape without the need for programming after printing, which is essential in common 3D printing. By incorporating the abovementioned materials in a tailorable fashion, the final synthetic products will have different mechanical strengths, even allowing each part to have a specific function.

Interestingly, the idea of this technology comes from natural samples. For example, the nastic plant motion of flowers and leaves occurs in response to external stimuli such as humidity, light, and mechanical pressure [252]. Furthermore, based on dynamic alteration in morphology and conformation of plant cell walls in response to environmental stimuli, Lewis and colleagues developed a novel programmable 4D printing system capable of creating complicated 3D structures by soaking in water [253]. The bio-ink used in this work was rigid cellulose nanofibrils incorporated in the acrylamide matrix, while nanoclay particles helped the viscoelastic features of ink. Due to the anisotropic swelling ability of such inks, they are named active composite materials [254]. Moreover, 4D printing technology has enabled the manufacturing of single-material completely soft robots, which can adjust their internal stress by changing their shape and main stress bearing point [255]. In recent decades, DNA origami has been opened as a new avenue to the design and synthesis of interesting automobile systems. Thanks to Japanese hand-working art, researchers began to use origami in 4D printing technology for establishing new self-folding and shape-forming complex 3D patterns [256].

### 5.1. Additive Manufacturing Technologies for 4D Printing

#### 5.1.1. Direct Ink Writing (DIW)

**Figure 2:**
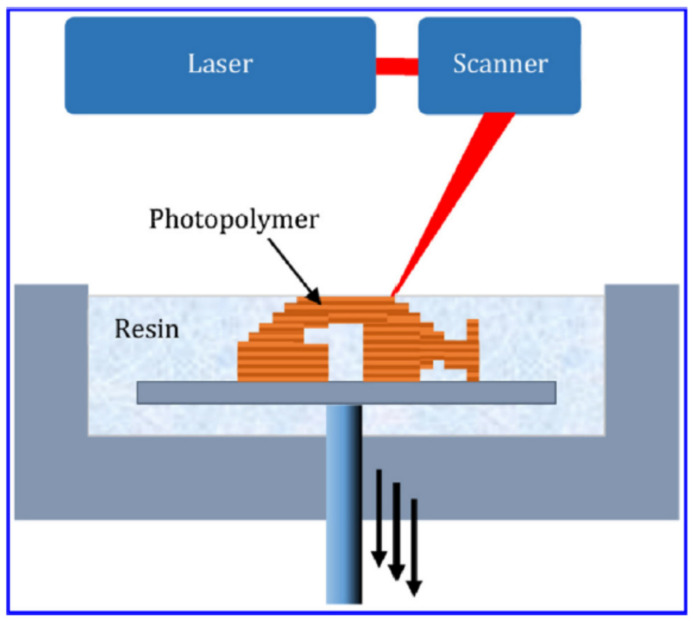
Illustrative scheme to introduce the configuration of stereolithography. Copyright 2016 John Wiley@Sons. Reprinted with permission from ref. [260].

One of the common drawbacks related to extrusion-based additive manufacturing (AM) is untenable mechanical properties due to their lower interfacial bonding. Achieving suitable rheological properties for better extrusion is one of the best strategies for extruding the viscoelastic polymer ink onto the stage from the nozzle [257] (Figure 2). By increasing the shear stress, following a decrease of viscosity, the internal pressure of the ink is significantly reduced. Furthermore, freezing the printed materials should be done precisely. DIW technique is used from UV to crosslinking the poly(lactic acid) (PLA)-based 4D architectures [258]. The best option for use as a solvent, in the case of PLA, is dichloromethane extruded from a micronozzle. Afterward, the solvent is evaporated, and UV is used for crosslinking process.

A thermoresponsive 4D structure can change shapes by repetitive heating and cooling above and below the PLA glass temperature of PLA (74.5 °C). Adding some of the aforementioned particles, like Fe3O4 nanoparticles, improves the mechanical strength and shape recovery ability. The glass transition temperature (Tg) is a pivotal feature that dictates the suitability of conjugated polymers for the application of different kinds of constructs. The Tg predicts the transition of the conjugated polymer into a brittle, glassy state, which directly demarks the soft, stretchable, or flexible electronics. The effective mobility value, ζ, is an atomic mobility value within each repeated unit in the polymer. This value is strongly related to the Tg of polymers [259]. Therefore, the Tg of every polymer used in 3D printing is a crucial factor for the reusability and recoverability of ink.

#### 5.1.2. FDM

Fused filament fabrication (FFF) is another sub-category route of DIW, called FDM. In this strategy, inter nozzle solid filaments are heated and lead to a softened extrusion. An ideal polymer for FDM is thermoplastic, which, analogous to DIW, melts and re-solidifies by cooling due to the non-covalently crosslinked nature of these materials. Some PLA strips can undergo pattern transformation thermally and have been developed by FDM polymer printers [261]. The synthesis method involves heating the PLA in the nozzle and then immediately cooling it below its glass temperature (Tg). By this technique, the reached pattern holds after elimination from the platform. Finally, pattern transformation occurred after reheating above Tg [255].

#### 5.1.3. SLA

For increasing the fabrication speed and spatial resolution, SLA was chosen as a vat polymerization AM process. Methacrylamides are used as photopolymerizable materials, and acrylates work as polymer resin for curing 3D structures with light [262]. Light source orientation dictates the SLA configuration; for instance, “upside-down” configuration results in a transparent window in its base. The “right-side-up” configuration leads to a printing layer with a thickness equal to the submerged depth of the build surface.

### 5.2. 4D Bioprinting of Cell-Laden Hydrogels

Another definition of 4D printing technology, which is different from the Gao description, is the usage of the bio-inks in the design of cell-laden experiments in which the interconnected cellular forces respond to intrinsic or extrinsic stimuli, resulting in a shape transformation [263]. Overall, 4D bioprinting, by a combination of stimuli-responsive bio-inks and novel cutting-edge 3D bioprinting techniques, created a new era for designing, manufacturing, and fabricating highly complex 3D bio-constructs. Obviously, these novel strategies allow us to design and synthesize more precise bio-constructs for resembling the natural tissue and organ function in an ex vivo manner [27]. One of the first discovered materials responsive to external stimuli (pH) changes is poly (acrylic acid) (PAAc) [173]. The main problem with 3D hydrogels is their unwanted interactions with some drugs (like cancer medication), subsequent undesired release profile, and off-target effects. Therefore, choosing the proper hydrogel for specific applications is an essential factor in the success of an experiment [264,265]. Among the polymeric materials, natural ones like chitosan, dextran, etc., suffer from poor mechanical features and consistency in biological media, which chemical modifications can help in some cases [266]. Instead of natural hydrogels, synthetic ones are better options because they do not have the aforementioned drawbacks, particularly biodegradable synthetic polymer polyesters.

## 6. Applications of 4D Printed Hydrogels

4D printing has been used in a variety of disciplines, and there is a lot of serious investigation on 3D printing with smart (stimulus-responsive) materials [267]. The 4D printed materials’ capacity to transform in response to external stimuli, as well as their time-evolving features, are interesting options for an extensive range of usages. Some key factors in biomedical applications, including biocompatibility, actuation temperature, and shape-changing rate, are typically taken into consideration. Physical features of smart materials, such as regulated electrical response, have also led to significant advancements in the electronics area [268]. This section discusses the various uses for 4D printing materials.

### 6.1. Drug Delivery

**Figure 3:**
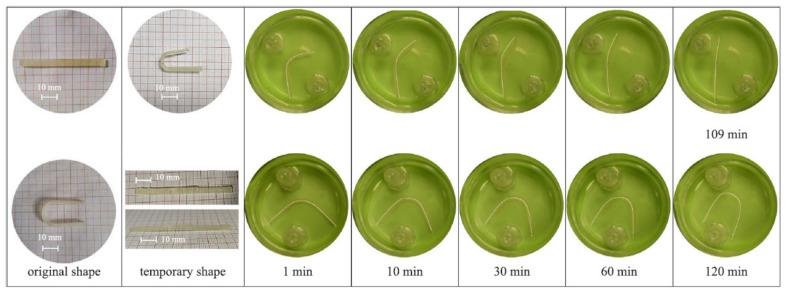
Images depicting the shape recovery process of the intravesical device (at around 20–22 °C) over time, which was created using FDM 3D printing and having a unique I- and U-form. To accentuate the recovery phase, a solid line is placed.

**Figure 4:**
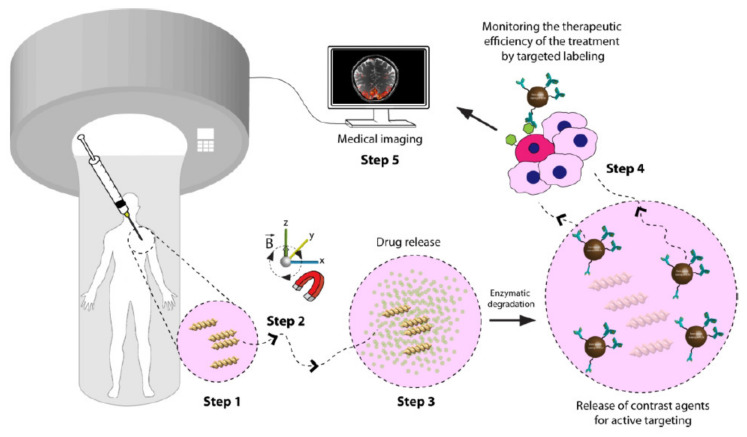
Step 1: Microswimmers are inserted into the tumor location’s vicinity. Step 2: Recruiting far-off magnetic powering and steering, functioning navigation, and exact localization of the microswimmers to the location of medicinal intervention. Step 3: Therapeutic intervention based on pathogenic signal input at the tumor microenvironment with controlled cargo release. The MMP-2 enzyme is shown to cause increased load release at pathological quantities, while physiological enzyme concentrations go undetected. Microswimmers are further degraded, allowing for maximal remedial load bioavailability. Step 4: Intact biodegradation allows the microswimmers to be safely eliminated and diagnostic contrast agents to be released. Step 5: Antibody-modified magnetic contrast agents permeate throughout the tissue to mark the untreated areas. The marked areas might then be evaluated in a minimally invasive manner to assess the treatment’s remedial efficacy and identify intervention places in the following phase. Copyright 2019 John Wiley@Sons. Reprinted with permission from ref. [275].

Because of the paucity, specificity, and multiplicity of clearance paths in an individual body, traditional delivery systems necessitate frequent and high dose administration to be successful [50]. In this discipline, adverse effects that could affect the entire system and decreased efficiency are major obstacles. The optimal drug delivery system must be able to reach the desired location in a controlled way and release pre-planned drugs, either independently or under supervision, based on environmental clues. Due to its potential to alter the form in response to exterior stimuli and alterations in ambient variables, such as pH and biological molecules, 4D-bioprinted hydrogels are being developed as a paramount classification of drug delivery materials [269]. To obtain desired dose profiles, pharmacological components are often incorporated inside the hydrogel medium and can have a controllable release by exterior stimuli-driven form alterations [270]. Sodiumalginate and pluronic F127 diacrylate macromer (F127DA) were recently combined to create shape-memory hydrogels (SMHs) [271]. The hydrogels had two network structures: a permanent network created by F127DA photocrosslinking and a reversible lattice-shape by Ca2+ crosslinked alginate. The use of SMHs as a medication delivery mechanism was made possible by loading them with MTX, a normally utilized anticancer agent. They evaluated the released medication data in vitro and discovered that the printed SMH released more drugs in the same amount of time as the regularly manufactured SMH. This was due to the interior grids created by 3D printing increasing the drug’s surface area. Furthermore, alginate, a form of polysaccharide, may provide nutrients to the printed structure, promoting cell proliferation [272]. Enhancing bioavailability is a pivotal factor in oral formulation. As a result, medications that are sensitive to high enzymatic activity and low stomach pH are frequently given as an enteric-coated tablet, which shields the drug and avoids premature drug release in the stomach while allowing for maximum absorption in the small intestine. As a result of the use of PH-responsive hydrogels, drug release in the stomach can be reduced while drug release in the intestinal pH can be augmented [33]. Larush et al. employed the DLP method to generate drug delivery systems, with pH and shape-dependent swelling triggering drug release from the printed items [273]. The authors of this work revealed how 3D printing could be used to improve the efficacy of traditional solid shapes of administration by managing drug release as a function of pH and the exterior zone while also manipulating the geometric criteria. As a result of the responsiveness of the printed material, drug release in oral delivery systems might be tailored, with the drug being released at a specific place in the gastrointestinal zone, due to variations in the system’s pH. Melocchi and colleagues have created retaining devices for intravenous drug delivery [274]. Engineered SMPs were used to hold a transient conformation that allows for bladder administration. When the devices come into contact with water, they revert to their original shapes, allowing them to be kept in the bladder (Figure 3). Increased intravesical residence time could improve treatment efficiency by allowing for extended, localized drug delivery. Ceylan et al. used the proteolytic degradation features of gelatin methacryloyl to make biodegradable microrobot-based hydrogels for earmarked drug administration, detecting the matrix metalloproteinase 2 (MMP-2) enzyme in the environment to release a medication [275]. As shown in Figure 4, after injection of microswimmers to the vicinity of the tumor, an external magnetic field could remotely control them to reach the exact location. This helps to reduce the damage of microswimmers to the surrounding healthy tissues. During the tissue remodeling process, MMP-2 destroys collagen proteins, which form the primary structure of the extra-cellular matrix. Although, tumor metastasis in a particular region will result in a rise in MMP-2 levels in that area, which is a pathological signal for microswimmers. As a result, the drug’s release will be accelerated by the enzymatic destruction of the hydrogel due to the pathologically high MMP-2 content. Following the enzymatic breakdown and full degradation of the structure, anti-ErbB 2 antibody-modified magnetic nanoparticles are released as a diagnostic contrast agent for labeling targeted cancerous cells. Furthermore, as the hydrogel deteriorates and releases the medication, hazardous remains are not left behind. In conclusion, findings show that 4D printing may be used to create structures that allow for drug localization and release rate control [276].

### 6.2. Tissue Engineering

**Figure 5:**
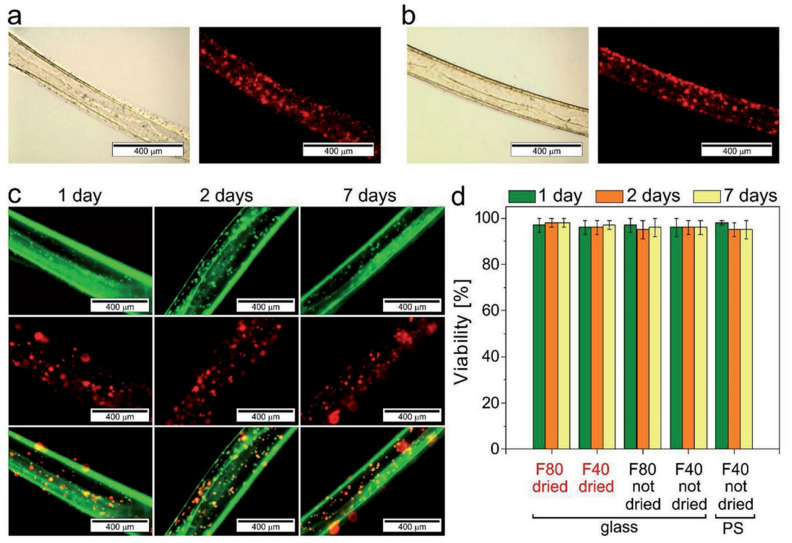
Four-dimensional fabricated cell-laden hydrogels. (**a**,**b**) Representative light (left) and fluorescence (right) microscopy images of self-folded AA-MA tubes; (**c**) fluorescent images of AA-MA tubes during different days; (**d**) cell viability assessment inside tubes during different days. The two types of samples (marked red) displayed good tube formation, and the other three samples (controls), which were printed without drying while crosslinking process, were provided as controls. Copyright 2017 Advanced Materials. Reprinted with permission from ref. [283].

Tissue engineering’s objective is to regenerate, restore, or substitute functional living organs and tissues that have been damaged or destroyed. Biomedical scaffolds constructed of original or synthetic polymers have been widely utilized in biomedical and tissue engineering usages to attain this goal [277]. Bioresorbable, bioactive, and mechanically strong biomaterials are characteristics of tissue and organ regeneration. The existence of 3D porous frameworks for cell ingrowth and matter transport, which allow new tissue creation and material biodegradation, makes scaffold-based techniques very promising for tissue engineering and regenerative medicine [25]. Biomedical scaffolds for tissue engineering necessitate a greatly porous 3D structure to perform these activities, which permits cell affinities to proliferate, migrate, adhere, and differentiate, as well as to transfer the nutrients and oxygen. Thus, 3D bioprinting is one of the best ways in which a 3D structure may be used for biomedical scaffolds, tissue, and organs [278,279,280]. Because of its soft, porous, water-bearing, and other extracellular matrix-like qualities, hydrogel is an excellent material for tissue engineering scaffolds. Hydrogels employed in tissue tailoring usages comprise alginate, gelatin, chitosan, poly ethylene glycol (pEG), and poly ethylene glycol diacrylate (pEGDA). Despite their ability to support complex biological structures, these hydrogels require dynamic and time-autonomous in vivo shape-changing behavior, as seen in the original tissues. Methods like 4D bio-fabrication and origami biosystems have been developed in recent years to imitate the complex structure and dynamic behavior of biological tissues and organs [27,281,282]. Kirillova et al. created a 3D-printed bi-polymer form-changing hydrogel by combining alginate, hyaluronic acid (HA), and bone marrow of mouse stromal cells, opening up new pathways to develop customized cell-laden form-altering frameworks for tissue tailoring [283]. The characteristics of these polymers can be further improved by utilizing appropriate compositions and including carboxylic groups in the chains to increase their sensitivity to Ca^2+^ ions for reversible shape alterations. Figure 5 a,b represents fluorescent microscopy and representative light images of these cell-laden tubes. Furthermore, the suggested 4D biofabrication approach has no negative impact on the viability of printed cells; the self-folding hydrogel-based tubes allow cells to survive for at least seven days without losing vitality, as shown in Figure 5c,d. Hydrogels’ ability to self-cure after mechanical damage makes them appealing for use in this area. In the presence of superparamagnetic iron oxide nanoparticles (SPIONs), Ko et al. showed that glycol chitosan (GC) and oxidized hyaluronate (OHA) are employed to generate a self-curing ferrogel without the usage of added chemical cross-linkers [284]. The GC/OHA/SPION ferrogel was magnetic field responsive, implying that it might be used in 4D printing. This biocompatible polysaccharide-based self-healing ferrogel could be beneficial in tissue engineering scaffold design and fabrication. Stimulus-sensitive hydrogels can be used to create a customizable and biocompatible environment with a variety of form and function-changing modalities. Gua and colleagues created nanoclay-incorporated double-network (NIDN) hydrogels via 3D printing [285]. Nanoclay interacts with polymer chains of methacrylated hyaluronic acid (HAMA) and alginate as physical crosslinkers (Alg). The findings revealed that the NIDN hydrogel system might be easily changed into a new kind of magnetic reactive hydrogel that supports the cell growth of bone marrow mesenchymal stem cells and has the ability to repair calvarial defects. Zhang et al. used self-fabrication of graphene oxide (GO) nanosheets, poly (vinyl alcohol) (PVA) chains, and G-quartet/hemin (G4/H) motifs to create a three-dimensional (3D) supramolecular hydrogel exhibiting stimuli-responsive characteristics in aqueous environments [286]. The GO/PVA/G4/H hydrogel had a honeycomb-like 3D GO lattice architecture and outstanding mechanical characteristics as it was manufactured. Encapsulating enzymes into hydrogels has also resulted in the creation of binary AND and OR logic circuits. The creation of unique “smart” soft matter for tissue engineering is now possible because of the development of multicomponent hydrogels. Repairing/regenerating diseased/defective osteochondral tissue is in high necessity as a result of the elderly society and the significant risk of sporty damages between youth. Because osteochondral tissue primarily comprises a structurally diverse and mechanically different subchondral layer and a cartilage layer, establishing a biomimetic bi-phasic scaffold with exceptional attachment vigor to regenerate osteochondral tissue is very required. Wang et al., for example, used cryogenic 3D printing to successfully build biomimetic osteochondral scaffolds with firmly linked subchondral and cartilage layers [287]. The subchondral layer had a hierarchically porous structure and mechanical properties that were identical to natural subchondral bone tissue, while the P(DLLA-TMC)/collagen I composite was able to imitate the structure and mechanical characteristics of native cartilage tissue. The presence of Tricalcium phosphate (TCP) and transforming growth factor-beta (TGF-1) increased and developed the osteogenic rat bone marrow-derived mesenchymal stem cells (rBMSCs) at both the subchondral and cartilage layers. Electrical stimulation is feasible to modulate cell response and maturation using electroactive hydrogels. As a result, they show potential in in vitro cell culture and tissue engineering using electrical agitation. Dister and colleagues described the 3D-printability of hydrogel precursors to build cytocompatibility and electrically conductive hydrogel scaffolds using pyrrole and a high gelatin-content oxidized alginate-gelatin (ADA-GEL) hydrogel [288]. The electrical/mechanical characteristics, 3D-printability, and cytocompatibility of the hydrogels are also evaluated. In contrast to leveled 2D hydrogels, bio plotting can be used to create open-porous scaffolds that are electrically conductive and possess greater cell culture effectiveness in scaffold profundity. Herreros et al. exploited 3D printed models for cancer stem cell (CSC) investigations to overcome the constraints of 2D in vitro cell seeding and laboratory animals as the preclinical cancer model systems [289]. Their results showed that 3D printed models, particularly those that employ GelMA-PEGDA hydrogels as the major scaffold material, are useful in investigating lung cancer stem cells. The findings showed that 3D printed scaffolds present acceptable results in simulating tumor intricacy and regulating cancerous cells in 2D culture models in vivo. Badea and colleagues also present a designed 3D cell culture in which a synthetic protein (RGD-PDL) is embedded into the microfilaments of 3D-hydrogel scaffolds to change interactivity and distinctively influence the tissue-level structure of complicated cell populations in vitro [290]. These scaffolds enable the formation of tissue-like structures, such as nerve-like bundles, from cellular processes.

### 6.3. Wound Healing

**Figure 6:**
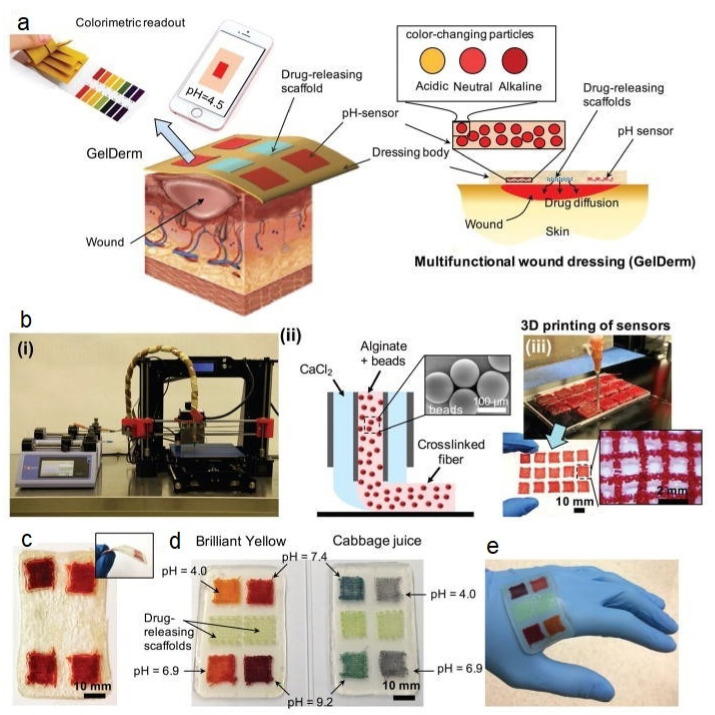
GelDerm is a sophisticated multipurpose dressing for wound monitoring and care. (**a**) A schematic of GelDerm’s epidermal wound therapy, featuring pH-sensitive and drug-eluting components. (**b-i**) A 3D bioprinter with a co-axial flow microfluidic nozzle was used to make porous sensors (i). (**b-ii**) Fiber deposition schematic employing a co-axial flow mechanism. (**b-iii**) A 3D printer can be designed to create porous sensor arrays for the manufacture of large-scale dressing. (**c**) Dressings can be lyophilized and sterilized for storage and transit. (**d**) Synthetic Brilliant Yellow and naturally generated cabbage juice were employed as model pH indicators for the manufacture of the sensors. Sensor arrays allow for the detection of spatial pH fluctuations at the wound site. Each time the dressing is removed, drug-eluting scaffolds release large dosages of antibiotics at the wound site, eradicating any germs that may have survived. (**e**) With uneven surfaces, GelDerm can maintain a conformal contact. Copyright 2017 John Wiley@Sons. Reprinted with permission from ref. [298].

**Figure 7:**
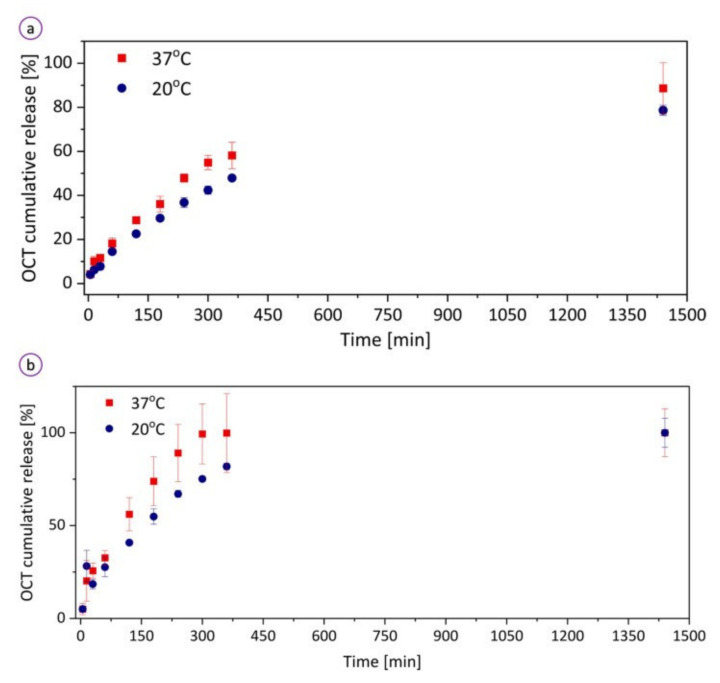
At temperatures of 20 and 37 °C, OCT release profiles from the hydrogel to (**a**) water and (**b**) PBS (pH 7.4). Copyright 2021 MDPI. Reprinted with permission from ref. [299].

**Figure 8:**
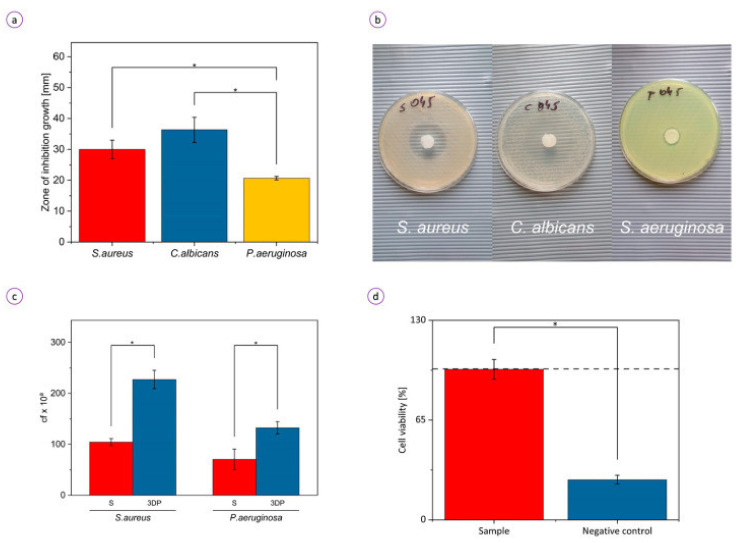
Biological activity of the hydrogel: (**a**) mediocre diameter of hindrance regions of *S. aureus, C. albicans*, and *P. aeruginosa* and (**b**) corresponding photos of the dishes; (**c**) ability of *S. aureus* and *P. aeruginosa* to colonize the molded (S) and 3D-printed (3DP) hydrogel samples. An exponent “a” corresponds to 9 and 6 for *S. aureus* and *P. aeruginosa*, respectively; “cf” means colony-forming; (**d**) viability of fibroblasts exposed to medium conditioned with elicits from the analyzed sample and ethanol (negative control). * *p* < 0.05. Copyright 2021 MDPI. Reprinted with permission from ref. [299].

**Figure 9:**
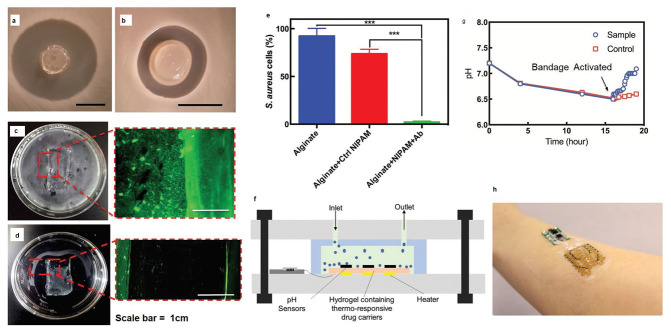
The antibacterial activity of the bandage was tested in vitro. (**a**,**b**) Antibacterial releasing hydrogel diffusion test and negative control (hydrogel without antibiotics). (**c**) On the control patch, live-dead due to biofilm development. (**d**) Live-dead staining on an antibacterial patch caused by biofilm formation; live bacteria appear as green. (**e**) Cefazolin-based CFU counting assay for *S. aureus*. (**f**) Schematics of an in vitro model for cultivating *S. aureus* bacteria in a bioreactor with a pH sensor and treated with an antibiotic patch. (**g**) In vitro test demonstrating pH variation over time, followed by heater activation at pH = 6.5. (**h**) The electronic component, pH sensors, microheater, and drug-loaded hydrogel are all integrated. The author’s hand was adorned with the patch; *** *p* ≤ 0.001. Copyright 2018 John Wiley@Sons. Reprinted with permission from ref. [302].

Wound management is a global concern, with the fast increase of chronic conditions such as diabetes, obesity, and the aging population, placing an enormous financial cost on the health care system. Wounds are a serious condition that can jeopardize the health of the skin. There are numerous hurdles to wound healing, including the presence of underlying illness, a massive volume of exudates, microbial contagion, inadequate perfusion, and poor nutrition [291,292,293]. However, this approach demands appropriate patient care and suitable wound coverage. Although standard wound dressings—e.g., gauze, lint, plaster, and bandages—protect the wound from pollutants, they require frequent alteration to refrain from neighboring tissue secretion, in addition to their propensity to stick to the wound, which makes their replacement painful [294]. New research on wound healing proposes a unique therapeutic method with the use of enhanced therapeutic possibilities based on 3D-hydrogel materials [295]. Hydrogel wound sizes have multifunctional features, including biodegradability, biocompatibility, continuous diffusion of bioactive compounds, high cell/drug embedding rates, a pore structure, and a suitable mechanical strength [296]. Alizadehgiashi et al. researched and introduced 3D-printed bio-composite hydrogel wounds that have been selectively loaded into the wound site with small molecules, metal nanoparticles, and proteins for controlled release at the wound site. To study the physiological response of the wound in a mouse model, hydrogel wound dressing, including antibacterial silver nanoparticles and vascular endothelial growth factor with pre-determined release profile, was used. The administration of dressings could improve granulation tissue formation and differential levels of vascular density, depending on the growth factor’s release rate, when compared to controls. This study highlights the potential of 3D-printed hydrogel dressings for establishing the optimal physiological responses in vivo, which can be tailored to treat a variety of wound types [297]. The pH of uninfected wounds is mostly in the acidic range from 5.5 to 6.5 during the healing phase, while the pH of infected wounds is more than 6.5. A bacterial contagion is often assumed to be present when the pH of a wound shifts from acidic to alkaline values [276]. For regulated drug and wound therapy, Mirani et al. used 4D bioprinting based on hydrogel dressings as drug-loaded and pH-reactant alginate scaffold arrays (Figure 6) [298]. Based on pH-induced color change and swelling (pH level variation posed by the bacterial infection), this multifunctional dressing was capable of monitoring the infection level of the wound and antibiotic release at the wound site. This study exhibits the ability of the dressing to detect bacterial infections utilizing in vitro and ex vivo tests with accuracies comparable to commercially existing systems. In another work, Niziole et al. used 3D printing technology to create thermo-responsive hydrogel-based wound dressings containing an antibacterial ingredient [299]. A novel printable ink containing poly(N-isopropylacrylamide) (PNIPAAm) precursors, sodium alginate (ALG), and methylcellulose (MC), that is laden with a mixture of octenidine dihydrochloride and 2-phenoxyethanol (Octenisept^®^, OCT), possesses accurate printability and shape fidelity. At two temperatures (20 and 37 °C), the hydrogel’s physicochemical characteristics and drug release profiles from the hydrogel specimens to the external solution were determined. The OCT distribution into ultrapure water and the PBS solution was sustained in the release test (Figure 7). Figure 8 shows that the temperature-responsive hydrogel has antibacterial action against *Staphylococcus aureus*, *Candida albicans*, and *Pseudomonas aeruginosa* and was non-cytotoxic to fibroblasts. This hydrogel is a potential family of materials for controlling chronic and acute injuries caused by trauma, surgery, or diabetes because of its thermo-responsivity, biocompatibility, antibacterial efficacy, and controlled drug release [300]. Common wound dressings are nearly opaque and must be uncovered frequently to monitor the progress of wound healing. Frequent wound disruption not only raises the risk of wound degeneration but also makes it difficult to monitor the wound in real-time [301]. To address this unmet medical need, a transparent dressing material that allows for real-time wound care is being developed. For example, Mostafalu et al. developed a smart and automated flexible wound dressing with temperature and pH sensors built onto flexible bandages that monitor wound condition in real-time [302]. Furthermore, an electronically controlled flexible heater and a stimuli-responsive drug-releasing system comprised of a hydrogel loaded with thermo-responsive drug carriers are incorporated into the wound dressing to release the medications on-demand (Figure 9). A microprocessor is built within the dressing to process the data collected by the sensors and program the drug release strategy for personalized treatment. This adaptable, intelligent wound dressing system has the potential to change the way chronic wounds are currently treated.

### 6.4. Vessel Response

**Figure 10:**
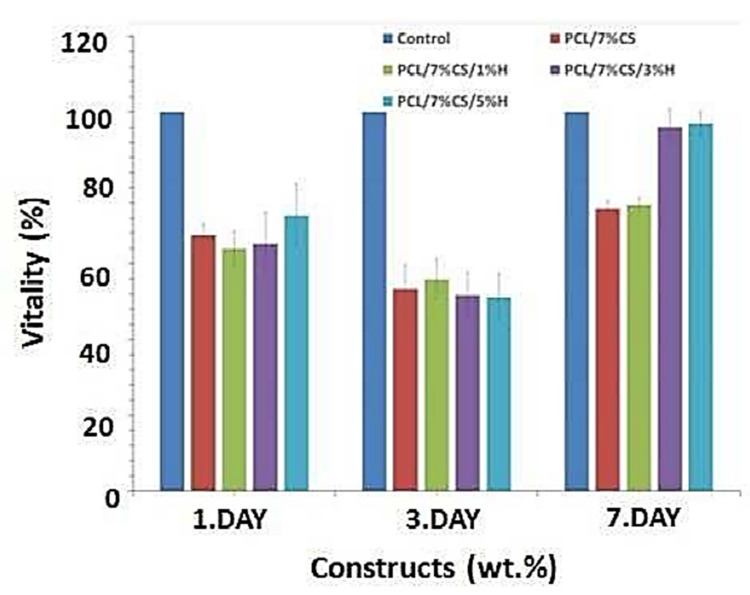
Results of cell vitality for all constructs showing on day 7, cell growth of PCL/7 wt.% CS/5 wt.% H was similar to the control group. Copyright 2019 John Wiley@Sons. Reprinted with permission from ref. [305].

**Figure 11:**
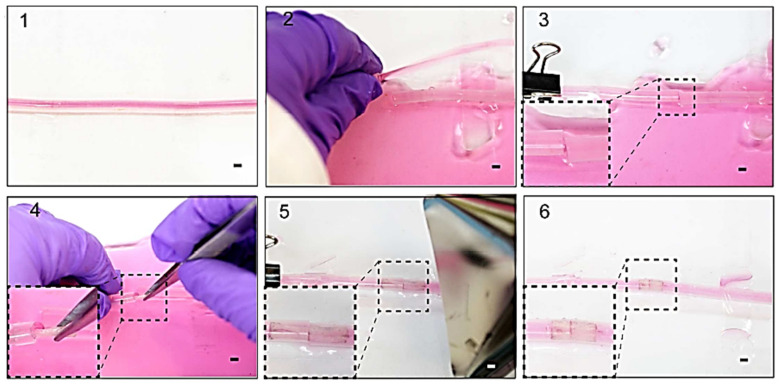
SM tubing for fast vascular reconnection and repair. The blood vessel (**1**) was cut for surgery (**2**); the severe “bleeding” was temporarily stopped by clamps (**3**); the broken “vessel” was implanted with the SM elastomer in the crack position (**4**). After being heated, the SM tubing returns to its original shape and is joined to the inside surface of the vessel (**5**). The fractured vessel was reconnected, allowing blood to flow (**6**). The scale bars are all 2 mm. Copyright 2019 ACS. Reprinted with permission from ref. [306].

Some of the most standard clinical treatments are vascular replacement and repair for the treatment of atherosclerotic disease, infection, and traumatic injury. Every year, hundreds of thousands of coronary and peripheral arteries are repaired, replaced, or bypassed in the United States alone. However, despite the tremendous clinical demand for engineered arterial replacements, the concomitant problems of thrombogenicity, infection resistance, and the ability to repair, remodel, and contract constructed arteries are extremely problematic to achieve [268,303,304]. Ulag and colleagues used 3D printing technology to create vessel-like constructions made of Poly (-caprolactone) (PCL), low molecular weight chitosan (CS), and alginate-hyaluronic acid-collagen type I hydrogels to overcome the issues associated with past use of traditional grafts [305]. This biocompatible, biodegradable, and nonimmunogenic hydrogel provides endothelial cells with a three-dimensional substrate in which to grow. The amounts of the abovementioned materials (CS, Alginate, HA, and collagen) used for hydrogel synthesis completely affect the survival rate of cells, individually, which are cultured in this mixture, as measured by mitochondrial dehydrogenase activity (Figure 10). When paired with the patient’s endothelial cells, the results demonstrate that printed vessels may be effective for the development of functioning blood vessels. Kuang et al. used the DIW technique to print a highly flexible semi-interpenetrating polymer network (semi-IPN) elastomer with an embedded semi-crystalline thermoplastic polymer, which is an in vitro example for vascular repair [306]. For the 3D printing of an (semi-IPN) elastomer that can be stretched up to 600 percent, ink containing urethane diacrylate and a linear semicrystalline polymer was produced. To imitate the environment of a blood vessel, a silicone tube was used and filled with red fluid. Two corners of the silicone tube were constricted to simulate hemostasis after the silicone was broken into two pieces. The printed hollow tube was stretched into a small diameter shape smaller than the inner diameter of the silicone tube. The damaged tube was then restored to its original shape after 30 s of heat stimulation, displaying a self-expanding characteristic to fully contact the inner silicone tube (Figure 11). As a result, it showed significant promise for fast reconnecting and mending the disconnected blood vessel. Vascular grafts that can allow comprehensive substitution and preservation of the wounded vessel by the body would improve surgical reconstruction results [307]. Scaffolds made from native vessel components can enable native cells to recognize them biologically and imitate the original vessel’s mechanical features. To create an adequate biological environment, as well as structural support, Bracaglia et al. suggest the construction of a 3D-printable and degradable hybrid scaffold by mixing polyethylene glycol (PEG) acrylate and homogenized pericardium matrix (HPM) [308]. When compared to single-component PEG or HPM hydrogels, this bioactive hydrogel scaffold could promote a mild inflammatory response and endothelial cell proliferation. The expression of pro-inflammatory cytokines such as TNF-α and IL-1β was lower with hybrid hydrogels (*p* < 0.05). This reduction in expression of inflammatory cytokines may have a positive effect on enhancing the healing rate at the implantation site [309]. Finally, digital light projection (DLP)-based 3D printing was used to create models of newborn vasculature utilizing this generated hybrid hydrogel. The structural control attained with this unique biomaterial suggests that it could be a viable new tool in the creation and research of vascular grafts. To accomplish the bifunctional properties of vascular transplantation, one of the best preparation procedures for the triple-layered vascular scaffold is to mimic the three-layer structure of native blood vessels. Hu et al. used electrospinning and coaxial 3D printing to create a flexible triple-layered vascular scaffold (TVS) [310]. The inner layer of the TVS was made from polycaprolactone-collagen (PCL-Col). An egg white/sodium alginate (EW/SA) blend hydrogel was extruded to generate hollow filaments that wound around the surface of the inner layer in a circumferential direction. Finally, as the outer layer of TVS, PCL-Col nanofibers were wrapped on the surface of the hydrogel layer. Findings demonstrate that TVS had superior maximum tensile stress (UTS) [311], strain to failure (STF), predicted burst strength, and suture retention strength (SRS) when compared to natural blood arteries. Human umbilical vein endothelial cells (HUVECs) were used to test the biocompatibility of TVS, and the results showed that the cells could effectively connect to the graft surface while maintaining high vitality.

**Figure 12:**
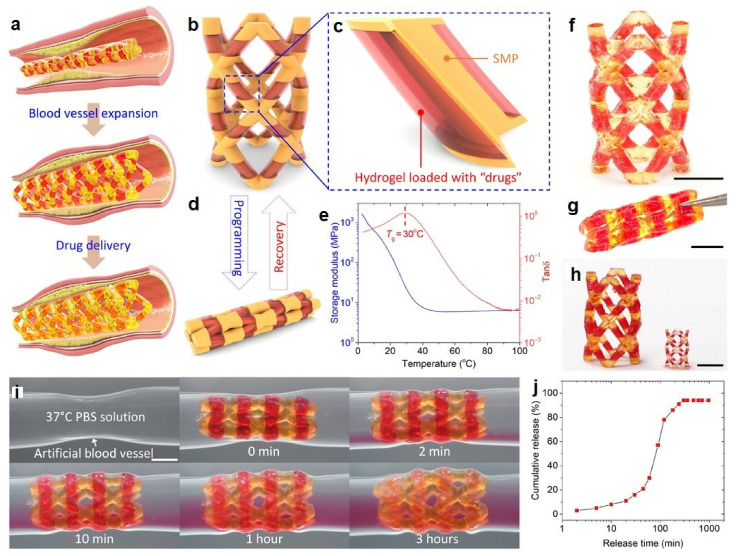
(**a**) Demonstration of blood vessel interaction with the 3D printed SMP-hydrogel stent and drug release profiles. (**b**) Overall design of the SMP-hydrogel stent. (**c**) The SMP rod is wrapped by a drug-loaded hydrogel skin, as shown in the detailed design. (**d**) SMP-hydrogel programmed to a compacted shape demonstration. (**d**) Illustration of the SMP-hydrogel programmed to a compacted shape. (**e**) DMA result indicates that the T_g_ of the SMP used to print the stent is 30 °C. (**f**–**h**) Snapshots of printed SMP-hydrogel stents. (**f**) As-printed SMP-hydrogel stent. (**g**) SMP-hydrogel stent in programmed compacted shape. (**h**) SMP-hydrogel stents with different sizes. (**i**) An example of both shape memory and drug release profiles. (**j**) Quantified drug-releasing process. Scale bar, 5 mm. (Photo credit: Jianxiang Cheng, Southern University of Science and Technology.). Copyright 2021 AAAS. Reprinted with permission from ref. [314].

Since its introduction to the medical field in the early 1980s, stents have progressed greatly in terms of their material, design, and structure. Drug-eluting stents (DESs) have been a game-changer in the treatment of coronary artery disease as compared to bare-metal stents (BMSs). In the field of cardiovascular stents, releasing pharmacological substances from the stent surface was a promising advancement [312,313]. Using 4D printing to integrate hydrogel into the SMP stent, Ge et al. created a cardiovascular stent with drug-releasing capabilities [314]. This hydrogel-polymer hybrid structure consists of extremely stretchy and high–water content hydrogels that are covalently coupled with different water-insoluble UV curable polymers to program the SMP-hydrogel stent at 37 °C, and the compressed shape may be fixed at 20 °C (Figure 12). Current commercially available stents are not degraded and remain in the body permanently, resulting in in-stent restenosis [315]. To address this issue, Veerubhotla et al. created a personalized biodegradable stent. The 3D printed stents were mechanically strong and robust when subjected to pseudo-physiological wall shear stress. The biodegradation rates of the stents were dramatically improved when nanofibers were added to the alginate hydrogel. These stents had no deleterious effects on human umbilical vein endothelial cells (HUVECs) or Raw 264.7 cells.

### 6.5. Electronic Applications

**Figure 13:**
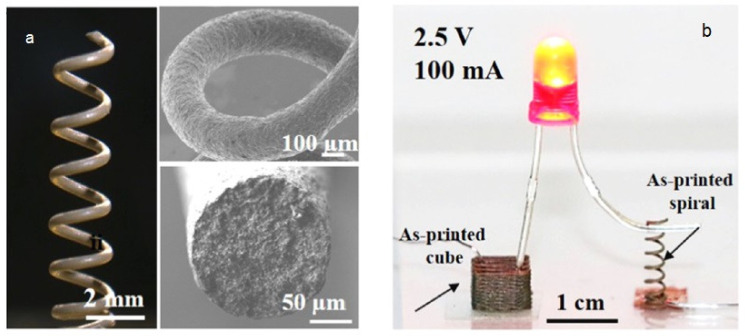
(**a**) Freeform 3D spiral. (**b**) Demonstration of the conductivity of as-printed structures, showing a cubic scaffold and a spiral connected to a circuit lighting up a red light-emitting diode (LED) under a voltage of 2.5 V with a current of 100 mA. Copyright 2019 ACS. Reprinted with permission from ref. [322].

**Figure 14:**
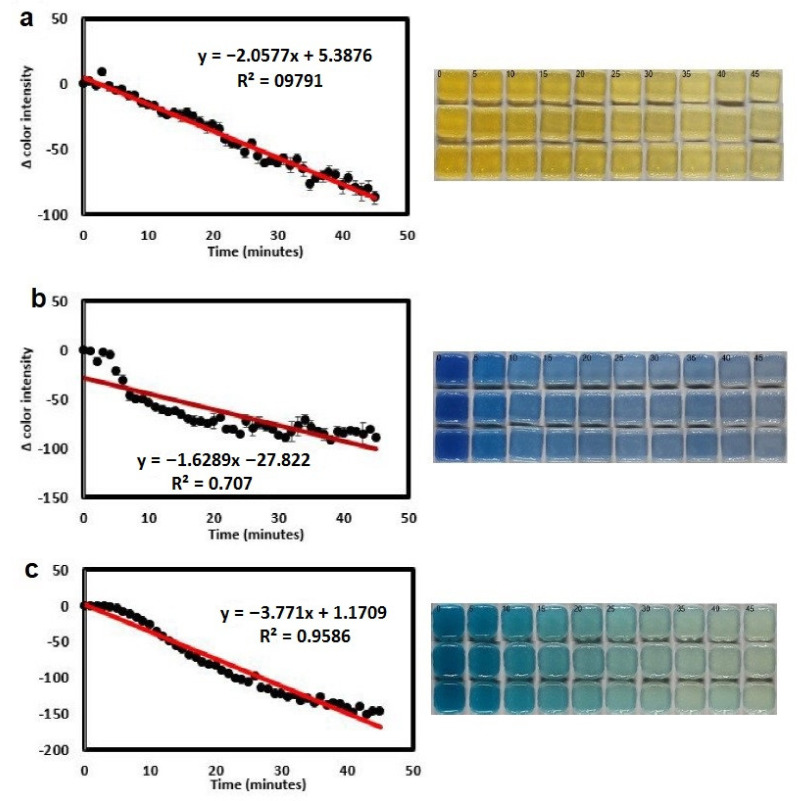
Colorimetric response showing discoloration resulting from the photocatalytic degradation of the three dyes following 45 min exposure for sensors for methyl orange (**a**); %RSD Minimum = 2.80, Maximum = 12.54, Mean = 6.15, methylene blue (**b**); %RSD Min = 3.18, Max = 14.21, Mean = 6.83, and malachite green (**c**) sensors; %RSD Min = 0.26, Max = 17.60, Mean = 3.14. Copyright 2020 ACS. Reprinted with permission from ref. [323].

**Figure 15:**
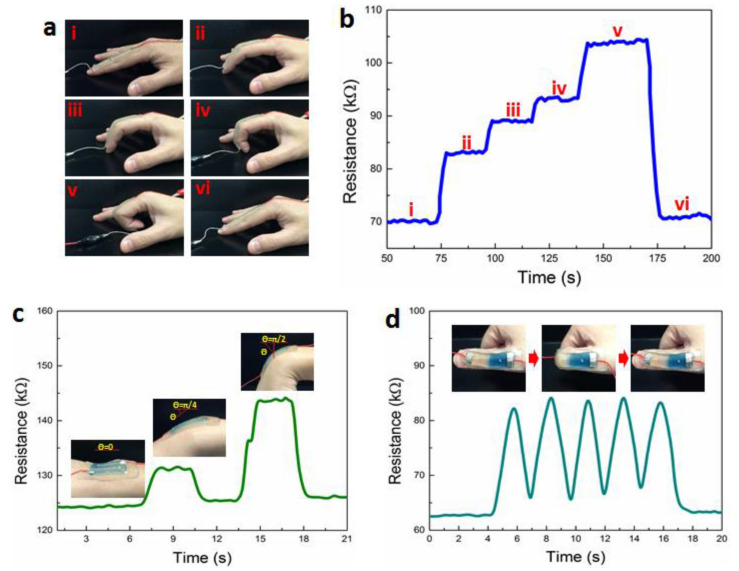
Real-time monitoring of numerous human motions (**a**), (**a**) the κ-carrageenan/PAAm DN hydrogel-based strain sensor fixed on forefinger to monitor its bending, and (**b**) changes in resistance as a function of time. (**c**) Change of resistance with time when the 3D printed DN hydrogel sample with the shape of number “S” was used as a conductor. The insets show the 3D printed DN hydrogel-based strain sensor attached to the wrist at different bending radians, 0, π/4, and π/2, respectively. (**d**) Change of resistance with time when the self-healed DN hydrogel was used as a conductor, and the insets show that the self-healed DN hydrogel-based strain sensors attached on the thumb finger bending repeatedly. Copyright 2017 ACS. Reprinted with permission from ref. [325].

**Figure 16:**
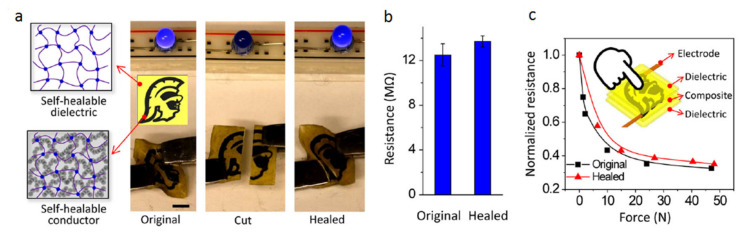
(**a**) A flexible Trojan pad with a self-healable elastomer phase and a self-healable conductor phase can power an LED. Once cut and healed after 4 h at 60 °C, the self-healed Trojan pad can again sustain bending and power the LED. The scale bar represents 4 mm. (**b**) The resistance of the conductive path of the Trojan path before and after self-healing. (**c**) The correlations between the original and self-healed force sensors’ normalized resistances and applied force. Copyright 2019 Springer Nature. Reprinted with permission from ref. [327].

Electroconductive 4D printed materials can be used in the field of electronics [316,317]. Parameters that play a significant role include voltage, electrical conductivity, and mechanical properties. Sensors are one example that uses conductive composites with 3D and 4D printability [268,318,319]. By integrating a 3D printed thermo-responsive hydrogel with submillimeter precision, Lei et al. produced a multifunctional and mechanically compliant skin-inspired sensor [320]. The double network hydrogels were made using a micellar copolymerization process in which hydrophobic n-octadecyl acrylate (C18) and N, Ndimethylacrylamide (DMA) were mixed in a NaCl aqueous solution to produce composite networks with physically crosslinked crystalline domains embedded in a covalent network. The smart skin is sensitive, stable, and demonstrates a very high-pressure sensitivity in 1 kP to sense body temperature, gentle finger touch, and finger flexing motion. The sensitivity of a hydrogel sensor (0–0.9 kPa) has a linear relationship from room temperature to body temperature. The temperature dependency of the sensor reveals a steady shift around 28 °C. Before the VPTT, the temperature might also enhance the skin-like sensor design for future artificial intelligence, wearable devices, and applications of man/machine interface [321]. Due to its promising electrical qualities, lightweight and tunable mechanical features, conductor polymer-composites (CPNs) are an essential class of functional elements in electronics. Wei et al. also printed highly conductive hybrid silver-coated carbon nanofibers (Ag@CNF’s)/PLA CPN [322]. The Ag@CNFs coupled the CNF’s high aspect ratio with Ag’s low contact resistance. There was a high-volume conductivity of >2 × 10^5^ S m^−1^ in printed nanocomposites. DCM was quickly evaporated, and the freeform 3D spiral could be printed at room temperature, as illustrated in Figure 13a. Figure 13b demonstrates that the LED can be directly driven by a power of 2.5 V with a current of 100 mA, exhibiting a strong conduciveness and readiness for use directly following the printing of the 3D-printed Ag@CNF/PLA nanocomposite structures. Excessive UV exposure can adversely affect human health. UV exposure may have harmful effects. Finny et al. produced the first portable, resilient, and economical 3D printed hydrogel sensors to detect UV exposure as colorimetrical indicators [323]. To construct them, independent structures were first printed using color-changing hydrogel ink. The ink contains alginate, gelatin, photoactive titanium dioxide nanoparticles, and dyes (methyl orange, blue methyl, and malachite green), in which the nanoparticles are utilized to photo-catalytically degrade the dyes and lead to dye discoloration [324]. The sensors are like a tattoo that changes its colors when exposed to UV radiation. The optimized technique allows mechanically stable sensors to be manufactured to detect outdoor sunlight effectively by quantifying the color decline, which is perceptible to the naked eye. Figure 14 illustrates the discoloration with 3D dyes and green malachite sensors made with three colored dyes. In addition to being utilized as wearable sensors, these sensors can be employed along with UV sterilization machines to assure the efficient exposure of surfaces to UV. Liu and Li have reported using the self-healing (SH) hydrogel as a stretchy printing sensor consisting of κ-carrageenan and polyacrylamide (Figure 15) [325]. The SH characteristics of the hydrogel were given by the thermo-responsive nature of κ-carrageenan. However, the SH only occurs when the dual helix of the material is separated into individual chains under high temperatures over the sol-gel transition point. SH was shown by re-joining two hydrogel fragments during 20 min of immersion in hot water at a temperature of 90 °C. The hydrogel can be employed as a sensitive strain sensor in robotics and human movement detection applications. A dielectric that allows electrical conductivity is the self-healing photo-elastomer [326]. Yu et al. used additive manufacturing to create a flexible composite pad with a dielectric and conductive elastomer phase and a University of Southern California (USC) Trojan contour path [327]. They exhibited that along the Trojan path, the sample is conductive and can power an LED, and at a large angle (~120°), it can be curved. The two sections were returned to their original shape to repair the interface after 4 h at 60 °C since both phases in the composite pad were self-healing. The healed pad reverts to being conductive and can be used to power the LED, but the resistance of the healed sample only changed by 9% (Figure 16a,b). To sense a signal to predict the applied force inversely, the ratio between relative resistance and recruited force can be utilized. The composite pad can be utilized as a self-healing force sensor as the conductive path’s strength diminishes when the compressive force increases (Figure 16c). This is due to the effective separation between black carbon particles in the conductor, which is smaller when a compressive force is applied.

### 6.6. Soft Robotic and Actuators

**Figure 17:**
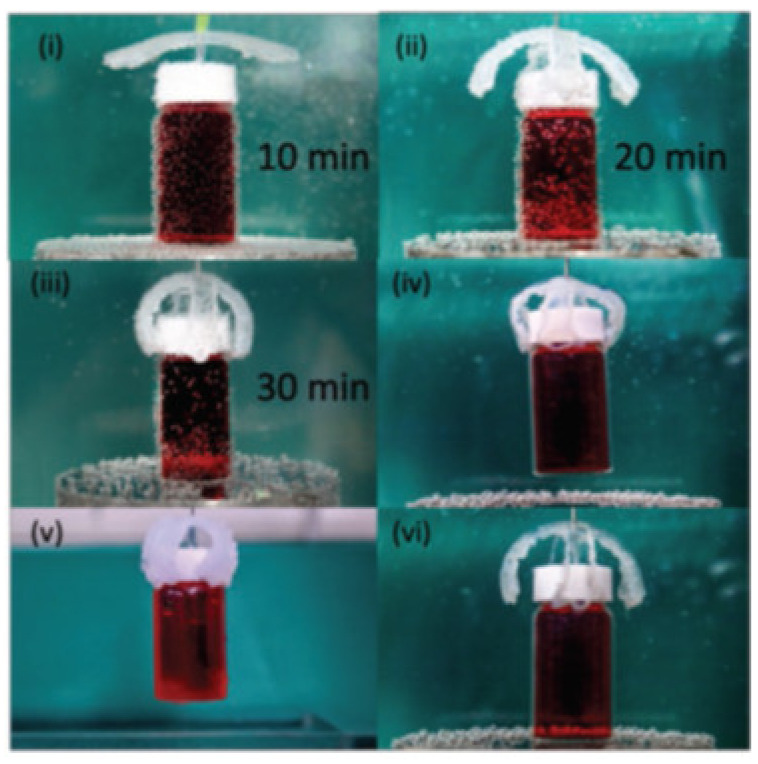
3D macroscopic gripper at different bending positions; (**i**) before gripping the vial; (**ii**) after gripping the vial; (**iii**) in the gripped state; (**iv**) lifting the vial in water at r.t.; (**v**) lifting the vial in the air at room temperature; and (**vi**) releasing the vial in water at 70 °C. Copyright 2019 John Wiley@Sons. Reprinted with permission from ref. [329].

**Figure 18:**
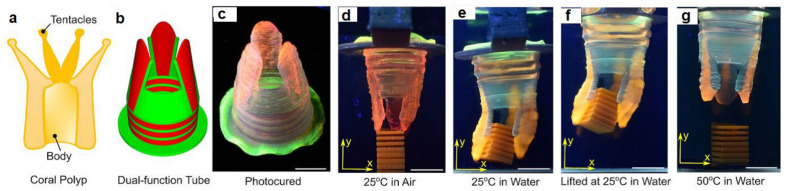
Dual-shape change tubes. (**a**) Scheme of the fundamental coral polyp anatomy; the picture was produced from encyclopedia polyp representations. (**b**,**c**) CAD model and 3D picture with cylindrical base, three fingers printed, and photo-cured tube. (**d**–**g**) Optical tube shapes alter at various temperatures. The tube has been suspended in a tank over a section. The tube displays uniaxial elongation and grasping of the portion when water was added to the tank. The tube shortened, and the fingers opened when heated to 50 °C to unload the component back down to the bottom of the tank. Scale bars are 1 cm. Copyright 2019 ACS. Reprinted with permission from ref. [330].

**Figure 19:**
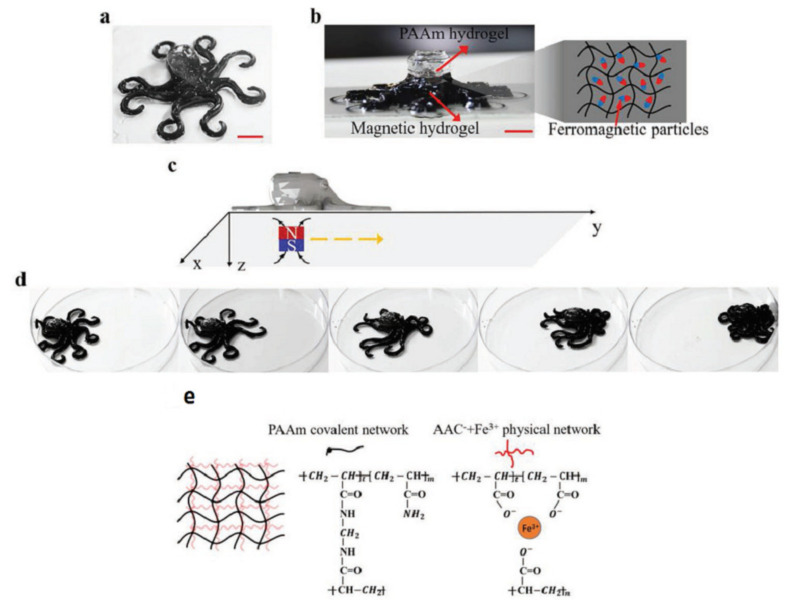
(**a**) Three-dimensional printed soft octopus hydrogels robot. (**b**) Front view of the octopus robot was manufactured in two components: (1) AAM-carbomer ink was used to print a transparent head, (2) AAm-carbomer ink was utilized for the printing of tentacles under the magnet field with ferromagnetic particles (Fe_3_O_4_). (**c**) Octopus robot scheme moving under a magnetic field driving on the “x-y” plane. (**d**) When the Magnetic Field is designed to travel from left to right, the octopus robot accomplished forward movement. (**e**) Schematic of the tough hydrogel. Scale bars, 1 mm. Copyright 2019 John Wiley@Sons. Reprinted with permission from ref. [331].

**Figure 20:**
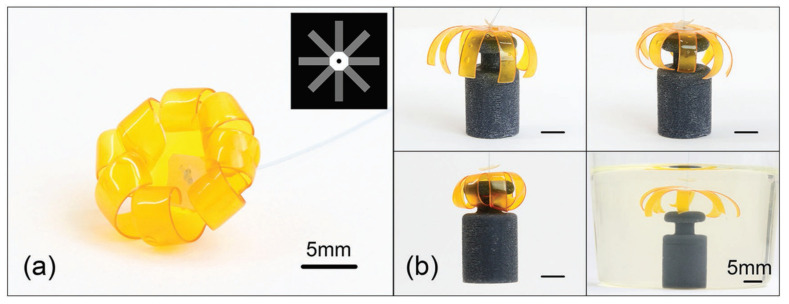
(**a**) Self-folding, flower-shaped gripper. (**b**) Grip- and release-process snapshots. The gripper was first swollen and opened in acetone (in around 10 s). The petals progressively folded with the volatilization of acetone after the opening gripper was placed on top of a weight (2.5 g) (within 2 min). Copyright 2016 John Wiley@Sons. Reprinted with permission from ref. [332].

Soft robotics has seen tremendous developments thanks to advances in the science of soft programmable materials, engineering design, and nonlinear numerical modeling. Soft robots are robots composed of flexible, soft materials inspired by nature and living organisms. Soft robots are required to handle and manage sensitive things on various scales for applications that would require secure human interactions, smooth textures, or in areas that are small and inaccessible [33]. Through self-folding or self-unfolding, as well as swelling/deswelling, in response to stimuli, the 4D printed grippers gain fine control to grab the object in a programmable manner [276]. The hydrogel-based gripper is suited for gripping soft items and has broad application in the biomedical field due to its softness, deformability, and ability to absorb shock [328]. Shiblee et al. created a gripper out of bilayer structures of shape-memory hydrogels manufactured by a proprietary stereolithographic printer (Figure 17) [329]. Within the shape-memory hydrogel (SMG) network, the bilayer was made up of poly (N, N-dimethyl acrylamide-co-stearyl acrylate) [P(DMAAm-co-SA)]-based hydrogels with different monomer concentrations of hydrophobic crystalline stearyl acrylate (SA). This model, with different physiochemical properties, has a floral architecture inspired by nature that changes shape when diving in water and is an underwater 3D macroscopic soft gripper capable of capturing, transporting, and releasing a guest substance. In another study, Liu et al. described thermoresponsive 4D printed bioinspired tubes capable of modifying their length, width, and bend according to the segmented models illustrated in Figure 18 [330]. The tubes consist of an active, thermal swelling gel (poly N-isopropylacrylamide; pNIPAM) and a thermal gel that is passive and non-responding (polyacrylamide; pAAM). It shows a structure inspired by coral polyps that can stretch and hold an object simultaneously [270]. In this field, magnetic reacting materials have also been developed. For example, Chen et al. made a magnetic hydrogel octopus with a forward movement that moves from left to right when the magnetic field is scheduled to “swim” through the petri dish (Figure 19) [331]. As shown in Figure 19a,b, the lower section of the 4D printed soft octopus robot is printed with magnetic hydrogel ink (the octopus tentacle), while the upper part is printed with transparent AAm-carbomer ink (the octopus head). The mechanical properties of the hydrogel were improved due to utilizing the AAm as a hydrogel base and interaction between the hydrogel matrix and carbomer polymeric chains. To construct three-dimensional electrical devices [332], Zhao et al. used digital processing technology and solvent-inducing self-folding. A photo-curable resin (poly(ethylene-glycol) diacrylate (PEGDA)) was printed in a flat sheet using a DLP printer, with the help of gray-scale light patterns and the addition of a photo-absorber (Sudan I) in the resin. The printed portions were solved by immersing the untreated oligomers in water and transforming the curated flat design into origami. Acetone is a suitable solvent that has solubility parameters close to a printed network. By adsorbing the solvent in a cross-linked PEGDA network, it swells and reshapes to a flat sheet. Figure 20a shows the flower-shaped, dissolved, pliable gripper, and Figure 20b shows images of the gripping action. The gripper was first opened by swelling in acetone (around 10 s). The petals were then placed on top of the weight (2.5 g), with acetone volatilization (within 2 min) progressively folding [319]. Actuators turn the environmental stimuli into mechanical movements and have been used extensively in nanomechanical, micro, and biomimetic systems [333]. In the field of actuator applications, stimulus-responsive materials have been highly enticed by the reversible change of form, which can be initiated by diverse external inputs. A number of actuators have been employed as hinges and valves in combination with 4D printing technologies [334,335]. Roach et al. used the hybrid print of liquid crystal elastomers (LCE), soft substrates, and conductive wires, which collected and positioned a ball when the current was on and off [336]. In this work, polycaprolactone (PCL) was used for the preparation of soft elastomer polymer resin. Hybrid printing processes were used to produce a soft gripper. Four LCE strips formed the hinges that displayed the bending behavior of conducting wires under the Joule effect. They also manufactured a robotic hand that imitates every finger bending where various areas were heated electrically. Hua et al. manufactured a hydrogel dual-responsive actuator using the hydrogel complex PAAm/PAAc [337]. A characteristic of the upper critical solution (UCST) temperatures may be exhibited by polyacrylic acid (PAAc) and poly(acrylamide) (PAAm) due to the creation of hydrogen connections from the polymer chains’ sections of PAAc and PAAm that have been widely researched in hydrogel UCST actuators. Due to internal water exchange between the two layers, temperature-driven self-actuation might take place both in water and oil baths. Auto-actuation with urea and salts may also be activated. The hydrogel formed by urea may disrupt the H-bonds between PAAc and PAAm. This new urea-induced alteration could, in form, result in potential material uses for bio-medical equipment [338]. This self-cure property can increase the duration and reliability of soft robotic systems [327]. The mechanisms of self-healing hydrogels are based on a cross-linking reversible interaction involving hydrogen bonding, supermolecular host guests, ionic, hydrophobic contacts, and covalent dynamic bindings [339]. Guo et al. designed highly flexible, ultra-tough, resilient glycerol hydrogels with self-healing features that are impressively tolerant to harsh conditions. They fabricated boron nitride nanosheet (BNNSs)/poly(acrylamide-co-maleic anhydride) (P(AM-co-MAH)) glycerol- hydrogels. The addition of (BNNS) and P(AM-co-MAH) to the hydrogel network results in a great self-healing capability. This structure shows remarkable dehydration resistance to high temperatures (60 °C). Furthermore, the presence of glycerol improves the anti-freezing feature of hydrogel composites in -45 °C conditions. Their high performance in a broad range of temperatures makes them a great candidate for use in different areas such as soft actuators [340]. In another study, Yu et al. produced soft actuators via photopolymerization-based additive manufacturing (AM) [327]. By applying negative pressure, the PDMS-based actuator can lift weights ten times heavier than its own weight. In this structure, dynamic disulfide bonds are used for rapid self-healing properties. So that, after halving the actuator, it recovered the initial structure and actuation ability by a disulfide metathesis reaction. Subsequently, the polymeric network self-healed at 60 °C for 2 h, and the self-healing process was sped up by rising temperatures [316].

## 7. Opportunities and Challenges of Smart Hydrogels for 4D Printing

Four-dimensional printing of hydrogel composites is an appealing method for additively manufacturing active structures for various electrical, mechanical, and medical applications. Nevertheless, 4D printing of detailed hydrogel-based devices is still challenging because of poor printability of hydrogels, and improvements to the mechanical performance and (bio)functionality are needed. This article presents a comprehensive review of recent trends, scenarios, and challenges in the 4D printing of hydrogels and hydrogel composites with an emphasis on their applications in tissue engineering, soft robotics, and biomedical devices. A brief overview of 4D printing methods of current hydrogel composites, including DIW, FDM, and SLA was provided. According to the types of additives, various hydrogel composite systems were discussed in this paper. Furthermore, emerging potential applications of hydrogel composites in the area of tissue engineering, soft robotics, and biomedical devices and their associated challenges were discussed in detail. Innovation in hydrogel composites, 3D printing, design, and modeling will create further progress in the 4D printing of hydrogel composites.
